# Enhancing the DNA yield intended for microbial sequencing from a low-biomass chlorinated drinking water

**DOI:** 10.3389/fmicb.2024.1339844

**Published:** 2024-05-24

**Authors:** Ratna E. Putri, Johannes S. Vrouwenvelder, Nadia Farhat

**Affiliations:** ^1^Environmental Science and Engineering, Biological and Environmental Science and Engineering (BESE) Division, King Abdullah University of Science and Technology, Thuwal, Saudi Arabia; ^2^Department of Biotechnology, Faculty of Applied Sciences, Delft University of Technology, Delft, Netherlands

**Keywords:** DNA yield enhancement, low-biomass sample, filter membrane, drinking water microbial community, reverse osmosis-produced drinking water

## Abstract

DNA extraction yield from drinking water distribution systems and premise plumbing is a key metric for any downstream analysis such as 16S amplicon or metagenomics sequencing. This research aimed to optimize DNA yield from low-biomass (chlorinated) reverse osmosis-produced tap water by evaluating the impact of different factors during the DNA extraction procedure. The factors examined are (1) the impact of membrane materials and their pore sizes; (2) the impact of different cell densities; and (3) an alternative method for enhancing DNA yield via incubation (no nutrient spiking). DNA from a one-liter sampling volume of RO tap water with varying bacterial cell densities was extracted with five different filter membranes (mixed ester cellulose 0.2 μm, polycarbonate 0.2 μm, polyethersulfone 0.2 and 0.1 μm, polyvinylidene fluoride 0.1 μm) for biomass filtration. Our results show that (i) smaller membrane pore size solely did not increase the DNA yield of low-biomass RO tap water; (ii) the DNA yield was proportional to the cell density and substantially dependent on the filter membrane properties (i.e., the membrane materials and their pore sizes); (iii) by using our optimized DNA extraction protocol, we found that polycarbonate filter membrane with 0.2 μm pore size markedly outperformed in terms of quantity (DNA yield) and quality (background level of 16S gene copy number) of recovered microbial DNA; and finally, (iv) for one-liter sampling volume, incubation strategy enhanced the DNA yield and enabled accurate identification of the core members (i.e., *Porphyrobacter* and *Blastomonas* as the most abundant indicator taxa) of the bacterial community in low-biomass RO tap water. Importantly, incorporating multiple controls is crucial to distinguish between contaminant/artefactual and true taxa in amplicon sequencing studies of low-biomass RO tap water.

## 1 Introduction

Freshwater scarcity is one of the major challenges that humanity faces; topped with the water pollution problem that needs to be tackled. Population Action International (PAI) has reported that at least 550 million people live in water-pressure or water-starved countries, and from 2.4 to 3.4 billion people will live in water-deprived countries by 2025 (Woo, 2018). To ensure a sustainable supply of fresh water and reduce the number of people suffering from water scarcity, membrane-based filtration processes have been developed to leverage the water supply worldwide (United Nations, 2015). Membrane-based filtration processes (e.g., reverse osmosis/RO, nanofiltration/NF) can be used to produce high-quality water, including desalination (of brackish or seawater), wastewater treatment, and rainwater harvesting. The major difference between various membrane technologies lies in the size of the ions, molecules, type of microorganisms, and suspended particles retained or allowed to pass via the membranes (Woo, 2018). Reverse osmosis is the most used desalting technology (e.g., 84% market share; Jones et al., 2019) for supplying water to water-scarce countries like Saudi Arabia (the largest producer of desalinated water with 7.9 million cubic meters/day, 22% of global desalinated water production), and other Gulf Cooperation Countries (Woo, 2018; Care Water Solutions, 2023). The water obtained after desalination has a high level of purification (2–10 ppm dissolved solids) due to the rejection of particulates, ions, molecules, and microorganisms and consequently, should be remineralized, for example by adding magnesium and/or calcium to be fit for domestic consumption (Woo, 2018).

The permeate and remineralized RO drinking water has very low microbial loads and nutrients (Meckes et al., 2007; Park and Hu, 2010; Kantor et al., 2019; Farhat et al., 2020), however, microbial (re)growth along the distribution network and building plumbing have been reported due to changes in operational and physicochemical factors (e.g., residual disinfectant depletion, stagnation, plumbing materials, biofilm slough-off, treatment breakthrough; Park et al., 2018; Stamps et al., 2018; Kennedy et al., 2021; Farhat et al., 2022). The conventional methods to monitor changes in microbial water quality of low-biomass RO-produced tap water usually fail to give a complete picture of the microbial dynamics. For example, a heterotrophic plate count and total coliform bacteria always resulted in below detection limit (e.g., <1 CFU/mL; Farhat et al., 2020) and at higher detection levels, mitigation of failure in the water distribution system is already too late (e.g., pipe check, stop water flow). To encounter this issue, the use of highly sensitive methods such as flow cytometry, and/or amplicon sequencing becomes imperative. Flow cytometry measurement for bacterial abundance can provide a (real-time) snapshot at a lower detection limit (e.g., 10^2^–10^3^ cells/mL; Prest et al., 2016; Van Nevel et al., 2017). Meanwhile, amplicon sequencing studies can provide information on the influence of process conditions and environmental parameters on the microbial community composition of drinking water in the network and premised plumbing (Pinto et al., 2012; Hull et al., 2019). However, there is a need to evaluate the robustness of DNA-based analysis for RO-produced drinking water.

One fundamental requirement in amplicon sequencing is to have a standard minimum concentration/yield of extractable DNA as a starting material. For example, Illumina recommends the amount of extracted DNA above 1.5 ng/μL for 16S rRNA amplicon analysis (Illumina, 2013, 2021). Any variations above or below such value depend on the school of thought (i.e., stop testing approach due to the low DNA, or enhanced interrogation approach analysis done by adding more replicates) of the laboratory operational standard or researchers’ objectives (Butler and Hill, 2010). Protocol optimization is, thus, suggested to overcome the low amount of DNA, such as: (i) increasing sampling volume that has been done in many documented studies (range from 100 mL to 1,000 L; Zhang and Liu, 2019; Bian et al., 2021), which has been proven not working for low-biomass drinking water samples produced by RO and/or NF for sampling volume up to 100 L (Stamps et al., 2018; Brumfield et al., 2020; Putri et al., 2021); (ii) adding multiple (negative and/or positive) controls to check for cross-contamination as low-biomass samples have been found to consist of bacterial community in the same range like those in controls/blanks (Salter et al., 2014; Eisenhofer et al., 2019); and (iii) ensuring that the detected microorganisms (e.g., bacteria) are the actual resident of the drinking water network/distribution system (La Duc et al., 2008). In practice, taking larger (e.g., hundreds or thousands of liter) sampling volume of membrane-treated drinking water like RO is questionable and has practical hindrance, given a high capital expenditure to produce the water. The range of bacterial cell concentration needs to be between 10^3^ and 10^5^ cells/mL to allow detectable DNA levels (Brandt and Albertsen, 2018) but chlorinated RO-produced tap water has a usual concentration of 10^2^–10^3^ cells/mL (Fujioka et al., 2018, 2019; Farhat et al., 2020). Furthermore, a lot of studies for bacterial community characterization in low-biomass samples such as disinfected drinking water known for very low DNA yield did not incorporate (negative) controls/blanks (Putri et al., 2021; Štiglić et al., 2023) on the premise that the identified community composition aligned with previous research from similar environments. Without negative controls, there is a risk of false positive findings and equally, propagation of false negative reports if done without confirmation of the actual bacterial community resident in low-biomass (and disinfected) membrane-produced drinking water like RO, that could be done for example by a simple incubation experiment (Countway et al., 2005). Therefore, this study aimed to evaluate the technical factors that affect the recovery of the extracted DNA concentration/yield from low-biomass (chlorinated) RO tap water. Specifically, the factors evaluated were: (1) different filter membrane characteristics (i.e., pore size and material), and (2) bacterial cell density regimes (pre-flush, post-flush, and incubated RO tap water) on a set of analysis outcomes (bacterial passage percentage, DNA yield, 16S gene copy number, bacterial community composition) typical for drinking water microbiome study. The following hypotheses were formulated: (a) DNA yield will proportionally correlate with biomass filtration efficiency; (b) a smaller pore-size filter membrane will produce higher DNA yield, and at least one filter membrane will outperform the others in terms of DNA recovery; (c) increasing cell density will give a higher DNA yield but could shift microbial community to some degree. To our knowledge, this is the first time a study has comprehensively compared all these factors, particularly for chlorinated RO-produced drinking water.

## 2 Materials and methods

### 2.1 Bacterial passage through different syringe filter pore sizes

**Figure 1:**
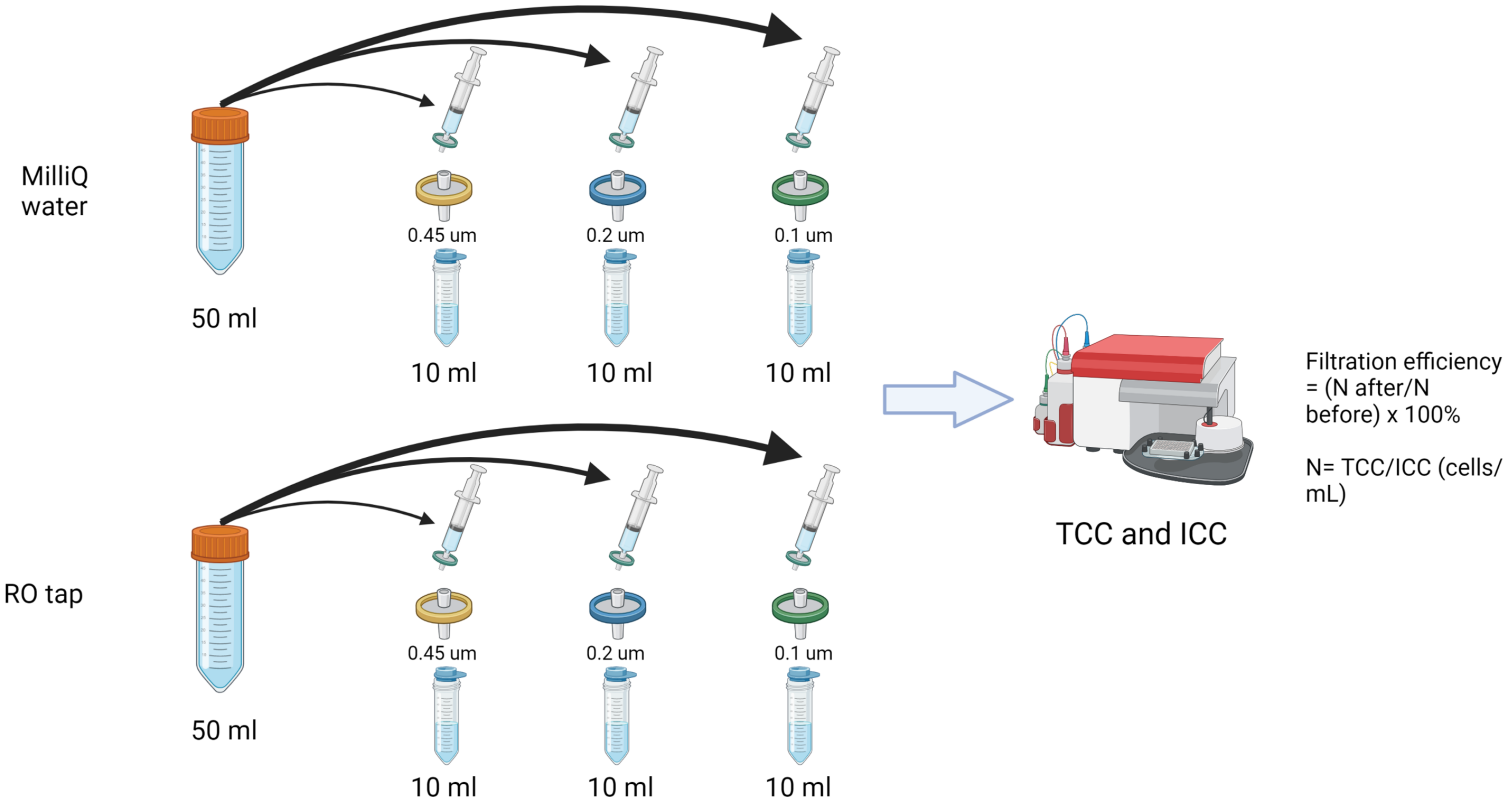
Schematic of the filtration efficiency experiment using commercially available syringe filters.

A syringe filter experiment was performed following a previously reported protocol (Wang et al., 2007; Figure 1). Commercially available (pre-sterilized) syringe filters with varied pore sizes were used: polyethersulfone filters (PES 0.2 and 0.45 μm) and polyvinylidene filters (PVDF 0.1 μm) after a preliminary optimization for testing different materials of syringe filters with the least background contaminant (Supplementary Figure S1; Supplementary Table S1). Evian bottled water served as a reference/control. Total and intact bacterial cell concentrations were measured in triplicates with flow cytometry-based staining using SYBR Green I and propidium iodide as previously described (Prest et al., 2013; Farhat et al., 2018). In short, 700 μL of water sample were mixed with 7 μL SYBR Green I for the total cell, or SYBR Green I (1:100 dilution in deionized water) and PI (4 μM final concentration) for the intact cell measurement. Stained samples were incubated for 10 min at 35°C in the dark and then measured with Accuri C6 Plus FCM (BD Biosciences, Accuri).

### 2.2 DNA extraction with various filter membranes

Designated premised taps (Water Desalination and Reuse Center, King Abdullah University of Science and Technology, Kingdom of Saudi Arabia) were used for sampling RO tap water throughout this study (Supplementary Figure S2). The RO tap water is produced by the KAUST seawater reverse osmosis plant with details on the process being described elsewhere (Farhat et al., 2020, 2022). The RO tap water is a mixture of RO permeate that is dosed with chlorine, CO_2_, and lime and then stored in a storage tank before being distributed throughout the KAUST distribution network. We sampled the final distributed RO drinking water from the premised taps that are part of the distribution network. A one-liter sampling volume was used for biomass filtration and subsequent DNA extraction and collected using an autoclaved narrow-mouth polypropylene bottles (Nalgene, NG-2203-0010) for each sample groups (with replicates). Sample groups analyzed were: control, pre-flush, post-flush, incubated, and Evian bottled water. Pre-flush samples were taken from the first liter of water with exact stagnation durations unknown. Post-flush samples were taken after 5 mins of flushing (network quality water). Incubated samples were post-flush water, dechlorinated, and incubated to reach 10^4^–10^5^ cells/mL with naturally assimilable organic carbon contents (no nutrient spiking) for 2–3 days at 30°C (temperature for bottled water heterotrophic bacteria). Evian bottled water was used as a reference with an average cell density of 10^4^–10^5^ cells/mL. All samples were filtered for biomass and subsequently, DNA extraction, with a minimum of two biological replicates processed (the number of replicates ranged from two to eight), except for the control (blank) group which consisted of a virgin sterile membrane, a membrane filtering 1 L of Milli-Q water, and a membrane filtering 1 L of sterilized (autoclaved), incubated RO tap water for each different membrane types. A total of 1 L × 63 samples were processed for DNA extraction.

Five types of filter membranes were used for biomass filtration: Mixed ester cellulose (MEC, 0.2 μm, Millipore, GSWP04700), Polycarbonate (PC, 0.2 μm, Isopore, GTTP04700), Polyethersulfone (PES, 0.1 μm, Sartorius, 2250943), PES Sterivex (0.2 μm, Merck, Sterivex, SVGP01050), and Polyvinylidene difluoride (PVDF, 0.1 μm, Durapore, VVLP04700). Except for PES Sterivex 0.2 μm which is a pressure sterile filter unit, each membrane had a 47 mm diameter and was sterilized (autoclaved) before use. Membrane (biomass) filtration was done using a peristaltic pump Masterflex I/P (Model 77602-30, Cole-Parmer, United States) connected with a Masterflex tubing (Model 6437-73) and sterilized (autoclaved and rinsed thoroughly with MilliQ) Swinnex Millipore Unit (47 mm diameter), except for the PES Sterivex filter. The tubing and Swinnex filtration head were fastened with an adjustable plastic clamp and Teflon tape to prevent leaking. The filtration flow rate was approximately 0.04 L/min. For the PES Sterivex 0.2 μm filter unit, a portable peristaltic pump (Vampire Sampler, Buerkle) was used at an approximate flow rate of 30–50 mL/min.

### 2.3 Modified DNA extraction protocol for RO tap water

DNA was extracted using the DNeasy PowerWater Kit (Qiagen, Germany) following (Brandt and Albertsen, 2018) recommendation. In-house modification of the optimized DNeasy PowerWater extraction method (Vosloo et al., 2019) for low-biomass water (10^3^–10^4^) cells/mL was adopted throughout this study that involved mechanical, enzymatic, and chemical lysis (Vosloo et al., 2019). The optimized protocol enhances the bead-beating cell disruption method from the DNeasy PowerWater kit by involving physical, chemical, and enzymatic lysis. In this study, a different bead-beater machine was used in comparison to the original protocol (Vosloo et al., 2019), i.e., MBB-24 Mini beadbeater (Biospec, United States), and thus 2 mins cell disruption time with 3,400 rpm was used based on the manufacturer’s recommendation to maximize cell lysis. The modified DNEasy PowerWater protocol was able to increase the DNA yield of Evian water (10^4^–10^5^ cells/mL) 6-fold (Supplementary Table S3A) and 9.6-fold compared to the DNA PowerSoil (Supplementary Table S3B), ensuring high DNA yield for the subsequent process (e.g., DNA purification) for amplicon library preparation. Furthermore, the PowerWater-modified has a less preferential bias in regards to low abundance OTUs detected and best captures the overall bacterial diversity compared to DNeasy PowerSoil kit based on our preliminary analysis (Supplementary Figure S6). Multiple blanks (as mentioned in section 2.2) and negative control (no template DNA/reagent) were processed in parallel for DNA extraction. It is also beneficial to spike the sample with known bacterial cell culture to test for DNA extraction efficiency (Brumfield et al., 2020; Knupfer et al., 2020). However, the effectiveness of the current DNA extraction method without such internal bacterial culture is also reflected in the resulting bacterial diversity (compared to DNAEasy PowerSoil; Supplementary Figure S6), indicating the current extraction protocol’s ability to open and recover genetic information from bacteria of varying hardness (La Duc et al., 2008).

### 2.4 Measurement of DNA yield

Extracted DNA concentrations were measured with a Qubit High Sensitivity assay kit (Invitrogen, Thermo Fisher Scientific, Germany) using a Qubit 4.0 Fluorometer (Thermo Fisher Scientific). Briefly, a volume of 190 μL Qubit working solution was mixed with 10 μL extracted DNA. The detection range of DNA concentration with Qubit HS assay is between 0.01 to 100 ng/μL. Total DNA yield was calculated based on the measured Qubit DNA concentration times the volume of the final elution buffer used (50 μL). All of the DNA extracts were sent to the DNASense Laboratory (Denmark) for sequencing and the size and purity of the sequencing libraries were checked with Tapestation 2,200 and D1000/High sensitivity D1000 screenTape assay (Agilent, United States) that produce the gel electrophoresis image of the DNA analysis. Samples with DNA yield below the detection limit were not processed for further analysis.

### 2.5 Controlled stagnation experiment and multimetric analysis

Two dedicated taps were chosen for this experiment: tap 1 and tap 2 (Supplementary Figure S2) which were subjected to two different stagnation periods: 14 and 30 days. After the intended stagnation periods were reached, samples were taken for pre-flush, post-flush, and incubated samples (see 2.2. DNA extraction with various membrane filters section). Samples were processed for multiple parameter analysis consisting of abundance and activity measures: total and intact cell concentration (TCC/ICC), Adenosine triphosphate (ATP), and 16S gene copy number.

TCC and ICC were done with flow cytometry-based quantification using SYBR Green I and propidium iodide (PI) staining as described above (see section 2.1). Adenosine triphosphate (ATP) analysis was measured using a luminometer according to the manufacturer’s protocol (Celsis Advance, Charles River Laboratories, Inc., United States). TCC and ICC measurements were done in technical triplicate and ATP was done in technical duplicate. qPCR analysis of the 16S rRNA gene was done by DNASense Lab (Denmark). Primer pairs, amplification conditions, and DNA standard used are described (see Supplementary Method S1).

### 2.6 Statistical analysis

To detect the significance of differences in each of the measured parameters (DNA yield, and additional parameters: TCC, ICC, ATP, 16S gene copy number) among different sample groups, a two-way analysis of variance (ANOVA) was carried out among the following fixed factors for each experiment: (i) *DNA extraction with various membrane filters*: “Group type/cell density” (five factors: control, pre-flush, post-flush, incubated, Evian), “Filter membrane type” (five factors: MEC 0.2 μm, PC 0.2 μm, PES Sterivex 0.2 μm, PES 0.1 μm, PVDF 0.1 μm); (ii) *controlled stagnation experiment*: “Tap location” (two factors: Tap 1 and Tap 2), “Group type/cell density” (four factors: control, pre-flush, post-flush, incubated). The data were previously checked for normality and homogeneity of variance using a visual check for homogeneity of variances (residuals vs. the fitted values) and the normal probability plot of residuals (Mangiafico, 2015; Holbert, 2022). All statistical analyses were done using R within the R studio environment (version 4.3.1). For post-hoc multiple comparisons, the Tukey Honest Significant Differences (TukeyHSD) test was used (Mangiafico, 2015).

### 2.7 Bacterial community analysis

All samples from the controlled stagnation experiment were processed for 16S rRNA gene-based high-throughput sequencing on a MiSeq platform (2 × 300 bp; Illumina, United States). Library preparation and sequencing were carried out by DNASense (Denmark) following their Illumina custom protocol (Illumina, 2013, 2021). The primer pairs covering V3-V4 (bV34A) regions of the bacterial 16S rRNA gene were used: [341F] CCTACGGGNGGCWGCAG and [806RB] GGACTACNVGGGTWTCTAAT (Klindworth et al., 2013). Up to 10 ng of DNA extract was used for PCR amplification (first PCR), and Illumina Nextera adaptors were added at the second PCR step which is necessary for sequencing. After quality control and bioinformatics processing, reads between 8,284 and 47,917 were considered satisfactory (parameter: filtReads <8,000). Rarefied counts of each OTU were used in all subsequent data cleaning and statistical analyses.

For the controlled stagnation experiment, a total of 22 samples were successfully sequenced and processed for multivariate analysis to determine the impact of the following descriptors: “taps location’, “group type/cell density” and their interactive effect on the bacterial community composition data using a two-way permutational analysis of variance (PERMANOVA). The statistical test was done with the Bray-Curtis distance matrix. The pairwise-adonis test was then performed for multiple comparisons to determine which group type (cell density) had differing bacterial communities. A differential abundance test using the Differential gene expression analysis based on the negative binomial distribution (DESeq2; Love et al., 2014; Weiss et al., 2017) was done to identify which taxa were significantly (the least *p-*value) enriched post-flush and incubated samples vs. controls. This statistical test yields information on the artifact taxa and shift of bacterial community following cell density enrichment.

### 2.8 Data availability

16S rRNA amplicon sequencing data can be accessed via the Sequence Read Archive (SRA) of the National Center for Biotechnology Information (NCBI): Accession number PRJNA1043459.

## 3 Results

### 3.1 Bacteria smaller than 0.2 μm syringe filter pore size predominate RO tap water

**Figure 2:**
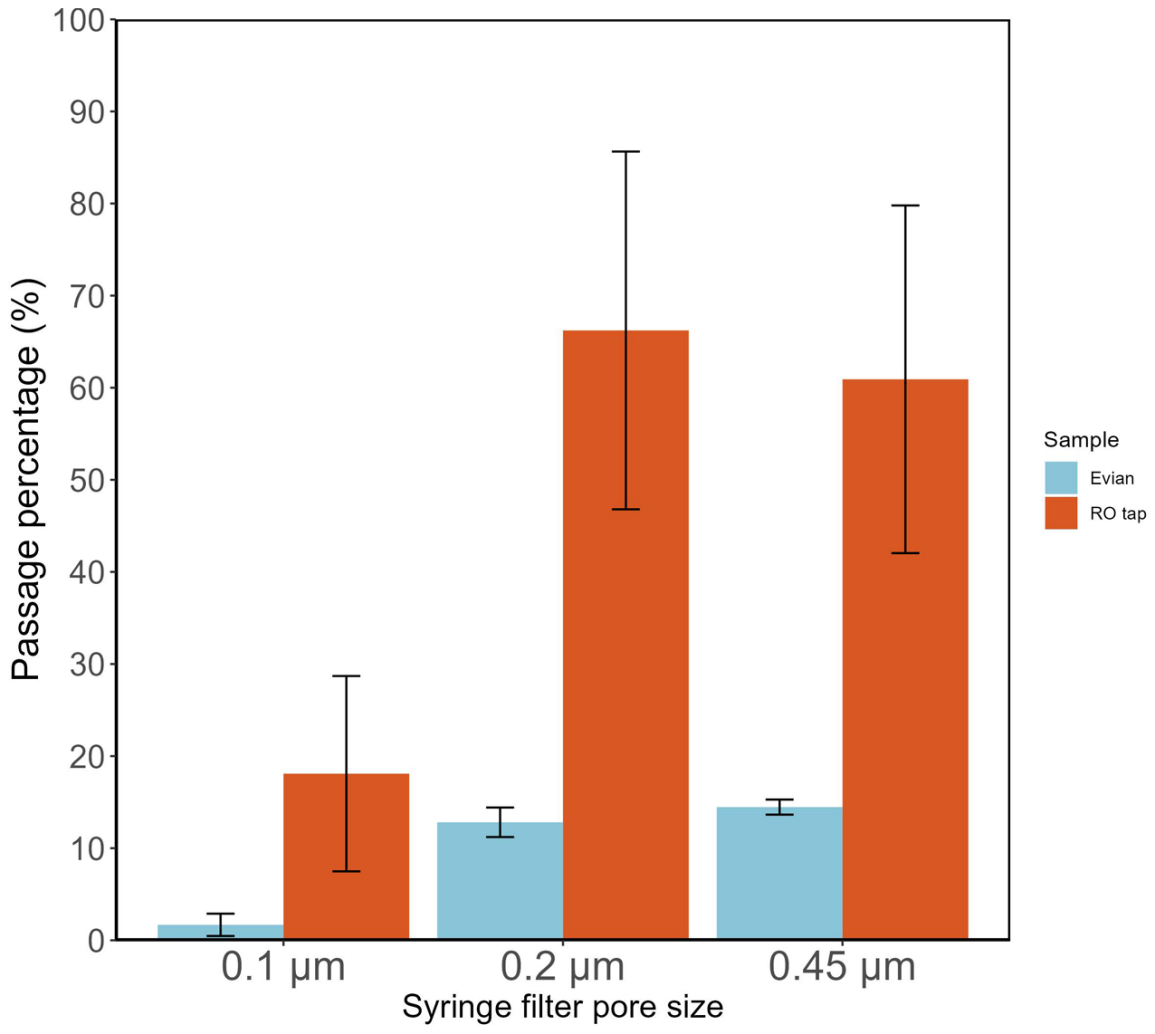
Fraction of intact bacteria passing different pore sizes (0.1, 0.22, 0.45 μm) of commercial syringe filters.

Measuring the percentage of intact filterable bacteria from RO tap water through various syringe filter pore sizes serves as a proxy test of biomass filtration efficiency of a particular filter membrane pore size. RO tap water samples had a high percentage (66.2%) of bacterial filterability through 0.2 μm pore size filters (Figure 2). This filterable fraction of intact bacteria was further reduced (18.1%) with lower pore size filters (0.1 μm; Figure 2). This fraction of bacteria is likely to reflect the population that survives chlorination in the network.

The influence of different syringe filter materials/manufacturers was tested (Supplementary Figure S1; Supplementary Table S1), showing that a certain type of filter material, i.e., cellulose acetate overestimated the actual bacterial count in the filtrate. These findings highlight the importance of the pre-filtration step before using a syringe filter in any subsequent analysis, e.g., microbial growth potential assay, to reduce the potential source of allochthonous carbon. Pre-washing a syringe filter with at least 200 mL of Milli-Q can remove contamination and reduce measurement error. Based on the results shown in Figure 1, it is reasonable to expect that DNA yield will be highly improved when using lower pore-size filters.

### 3.2 Impact of various filter membranes on DNA yield and 16S rRNA gene copy number

**Figure 3:**
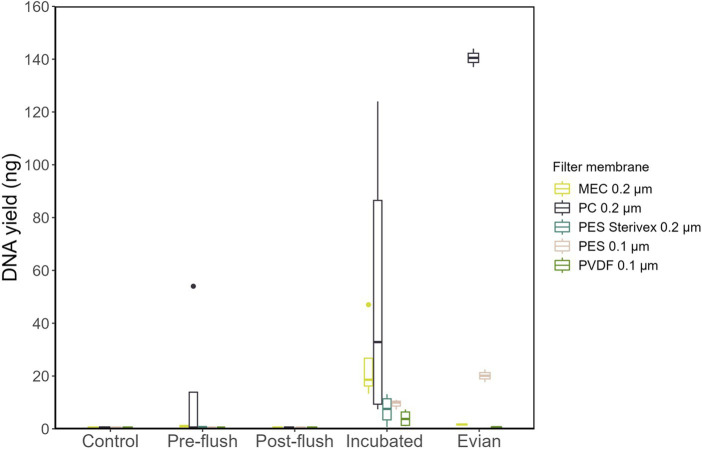
Extracted DNA yields obtained following extraction with different filter membrane types.

We investigated the technical factors influencing DNA extraction and yield enhancement from low-biomass RO tap water. First, to identify the optimal filter membrane type (material and pore size), a one-liter sample from each sample group (varied cell density) was filtered for DNA extraction. The sample groups included: pre-flush, post-flush, and incubated RO-produced tap water, Evian, and multiple controls. DNA concentrations were consistently low for post-flush and control samples (BDL < 0.01 ng/μL), irrespective of the filter membrane type used. Meanwhile, for higher cell density samples: RO-produced tap water and Evian, quantifiable DNA yields were obtained for both groups, 3.96–49.01 and 0.63–140.5 ng, respectively, across all filter membranes tested (Figure 3). The pore size effect, which is dependent on filter membrane material, showed no linear correlation with DNA yield, i.e., smaller pore size filters did not equate to higher DNA yields. Among different filter membrane types, significant differences in DNA yield were found (*p* < 0.05, Two-way ANOVA) and the polycarbonate (PC) filter membrane with 0.22 μm pore size substantially yielded more DNA compared to the other filter membranes tested (*p* < 0.05, post-hoc TukeyHSD test).

**Figure 4:**
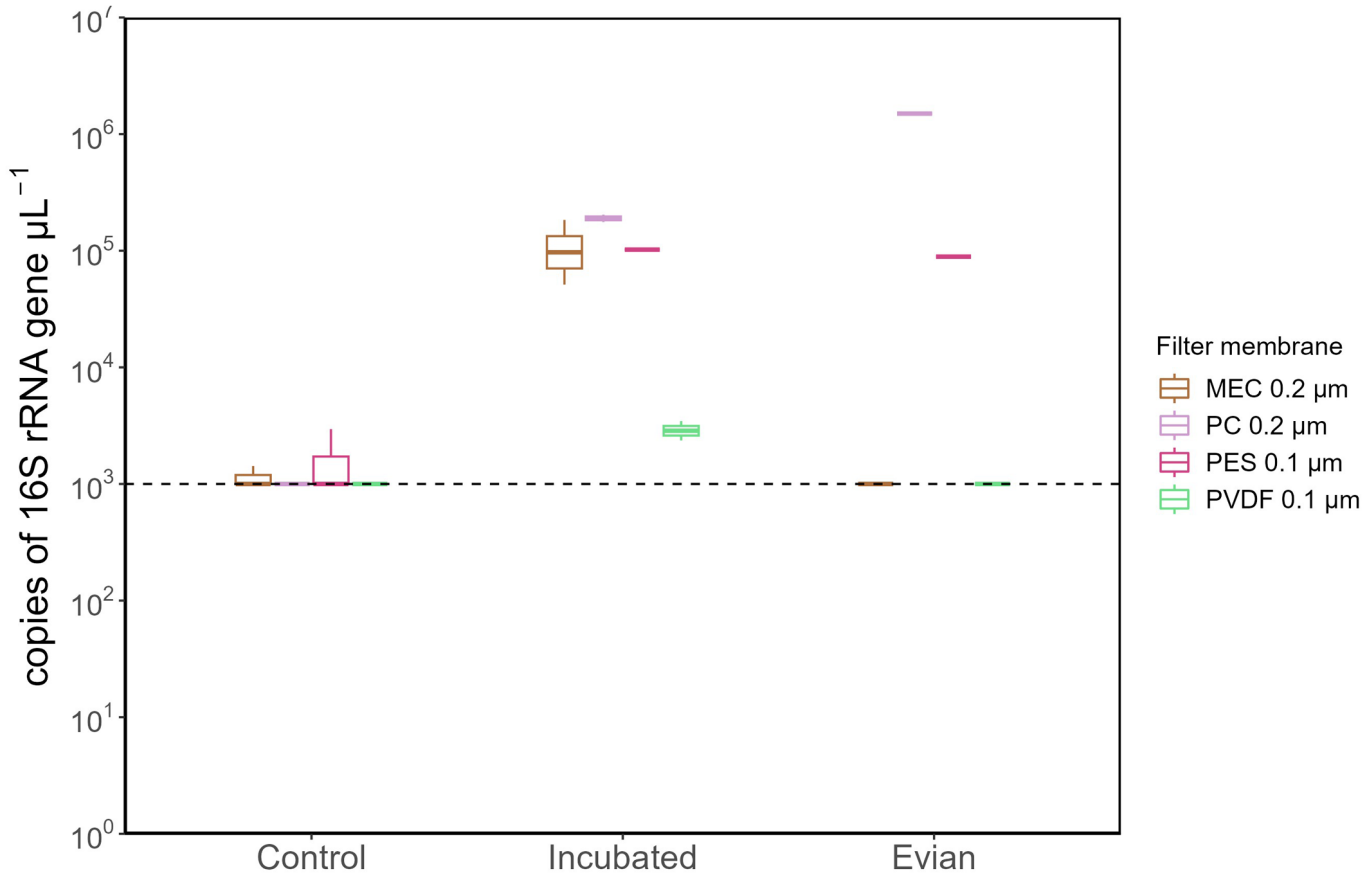
Total bacterial 16S gene copy numbers extracted with different filter membrane types. All samples from pre-flush and post-flush were below the detectable limit (1,000 copies/μL) and only samples from control, incubated and Evian were shown.

A subsequent analysis using qPCR was conducted to evaluate the total bacterial abundance (gene copy number/μL) in each sample group. All samples from the following: multiple controls, post-flush, and pre-flush, irrespective of filter membrane types had gene copy numbers below the detection limit (<1,000 copies/μL) and therefore not shown. Figure 4 shows the samples that had detectable gene copy numbers. MEC 0.2 μm and PES 0.1 μm filter membranes at control conditions had considerable background contamination in terms of 16S rRNA gene copy numbers (1,430 and 2,950 copies/μL, respectively, Figure 4). Meanwhile, PC 0.2 μm filters gave BDL at control conditions, corroborating the previous findings in DNA yield, and substantially resulted in higher gene copy numbers (Figure 4; *p* < 0.05, *post hoc* TukeyHSD test) for incubated samples and Evian water, both have higher cell density. From incubated samples, PC filter membranes enhanced the positive detection rate for total bacterial copy numbers with quantification values 158 times higher than controls. Overall, PC 0.2 μm pore size filters outperformed the other membranes in terms of the highest DNA yield and lowest background of 16S rRNA gene copy numbers.

### 3.3 Controlled stagnation experiment

Following the optimization of filter membrane type for enhancing the DNA yield, we designed the following controlled stagnation experiment to investigate the impact of increasing cell density on bacterial community composition, and the extent of its contribution to the set of parameters (TCC/ICC, ATP, 16S gene copies and DNA yield). DNA extraction was performed with PC 0.2 μm filter membranes only.

**Figure 5:**
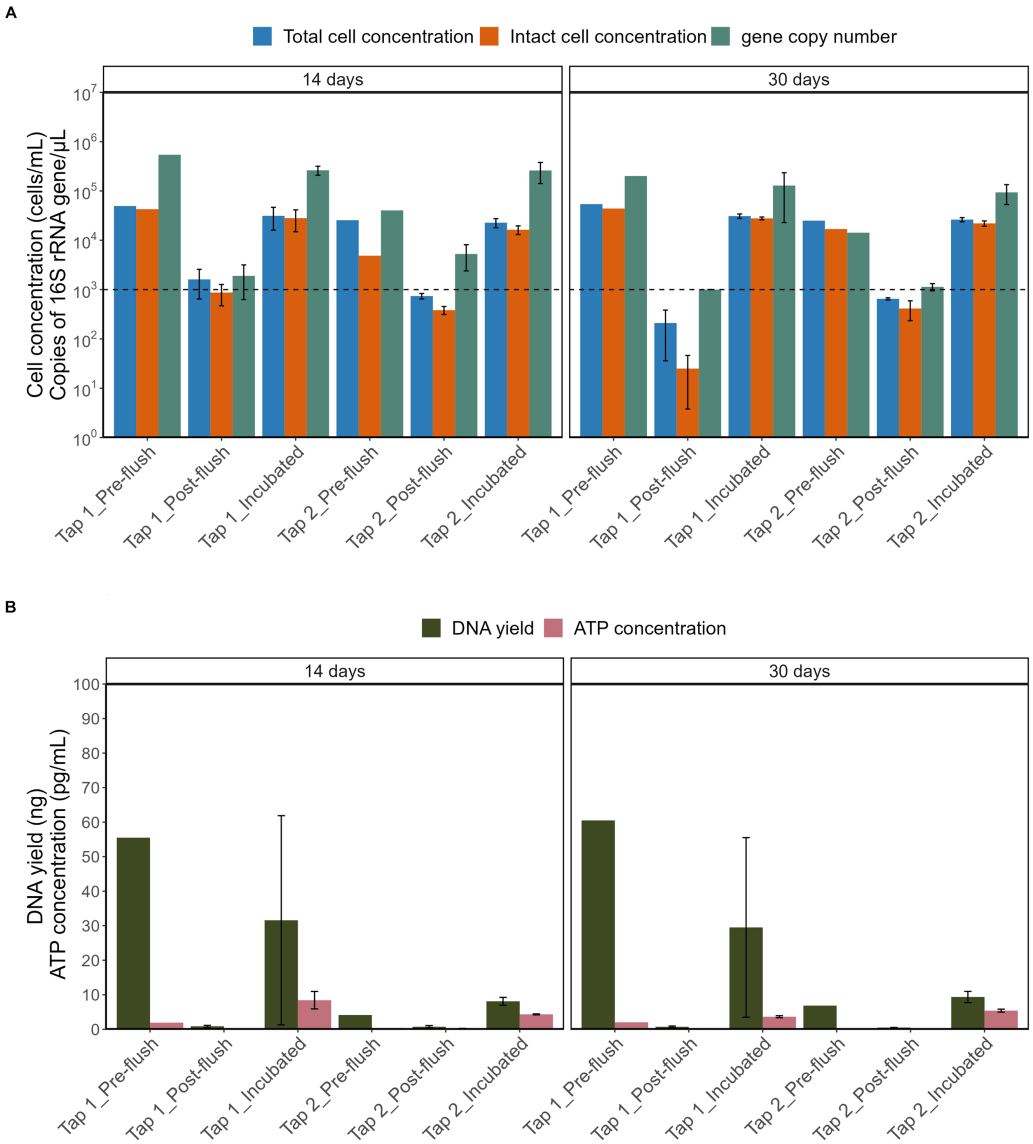
Comparison of **(A)** TCC, ICC, copy number of 16S rRNA gene and **(B)** DNA yield, ATP concentration across different sample groups. The horizontal line is set at method detection limits (flow cytometry at 10^3^ cells/mL and qPCR of 16S rRNA at 10^3^ copies/μL).

As shown in Figure 5A, stagnation had a similar effect on the TCC and ICC from the two tap locations: the maximum bacterial cell concentrations reached were 5-log [cells/mL; see Supplementary Table S1 for pre-flush and incubated samples while post-flush cell concentration is always consistent between 2- to 3-log (cells/mL)]. Consequently, these differing cell densities had substantially different DNA yields (Figure 5B), bacterial gene copy numbers, and ATP concentration (all *p*-values < 0.05, Two-Way ANOVA). Stagnation periods had no significant impact on the DNA yield obtained (*p* = 0.956, ANOVA). Pre-flush and incubated samples from tap 2 had overall lower DNA yields (6.85–9.35 ng) compared to tap 1 (29.5–60.5 ng) even though bacterial cell concentrations were within the same range with tap 1 [~4-log unit (cells/mL)] which is probably due to differences in DNA extraction efficiency and/or sample variations. However, even though differences in DNA yields were observed, the total bacterial gene copy numbers were similar for both taps [4.5-log–5.5-log unit (copies/μL)].

Our results emphasize that the lower bacterial cell concentrations in post-flush samples had significantly low DNA yield, and 16S rRNA gene copies (most are below the detection limit of 1,000 copies/μL) compared to the pre-flush and incubated samples that had at least 10^4^ cells/mL (all *p* values were <0.05, *Post hoc* TukeyHSD test). The consequence of these findings is that low-biomass RO tap water with cell density below 1,000 cells/mL will yield low DNA concentration, even after using a PC 0.2 μm filter that enhances DNA yield with minimum background bacterial copy number. Nevertheless, useful sequencing libraries were produced, and a straightforward incubation procedure evidently increased cell density, which is proportional to the higher DNA yield and higher total bacterial gene copy number.

### 3.4 Impact of increased cell density on the bacterial community profile

**Figure 6:**
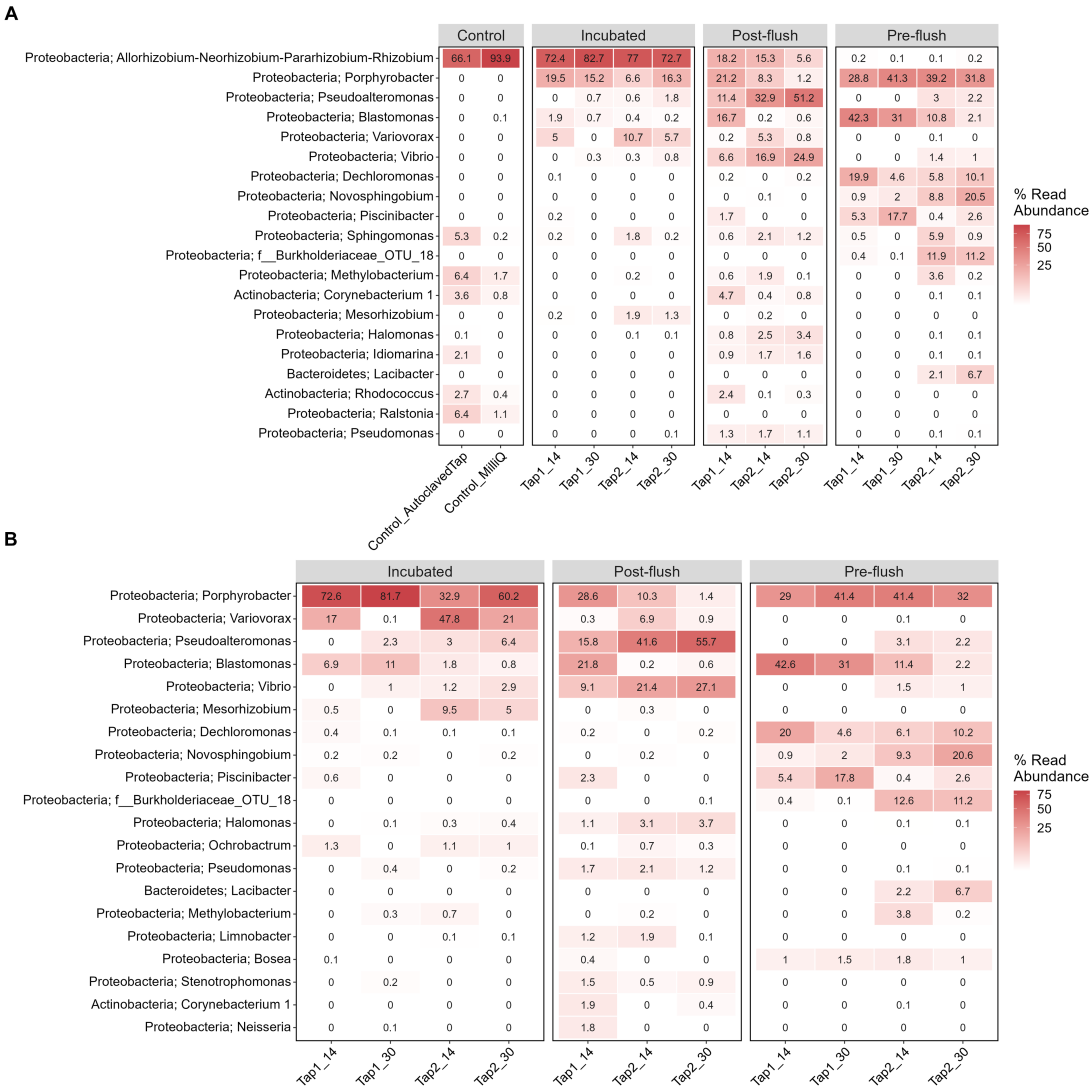
Heatmap of top 20 genera and their assigned phylum classification **(A)** before and **(B)** after data decontamination. Each column shows sample name, tap and stagnation periods. The number shows the relative read abundance from the average of sample replicates.

**Figure 7:**
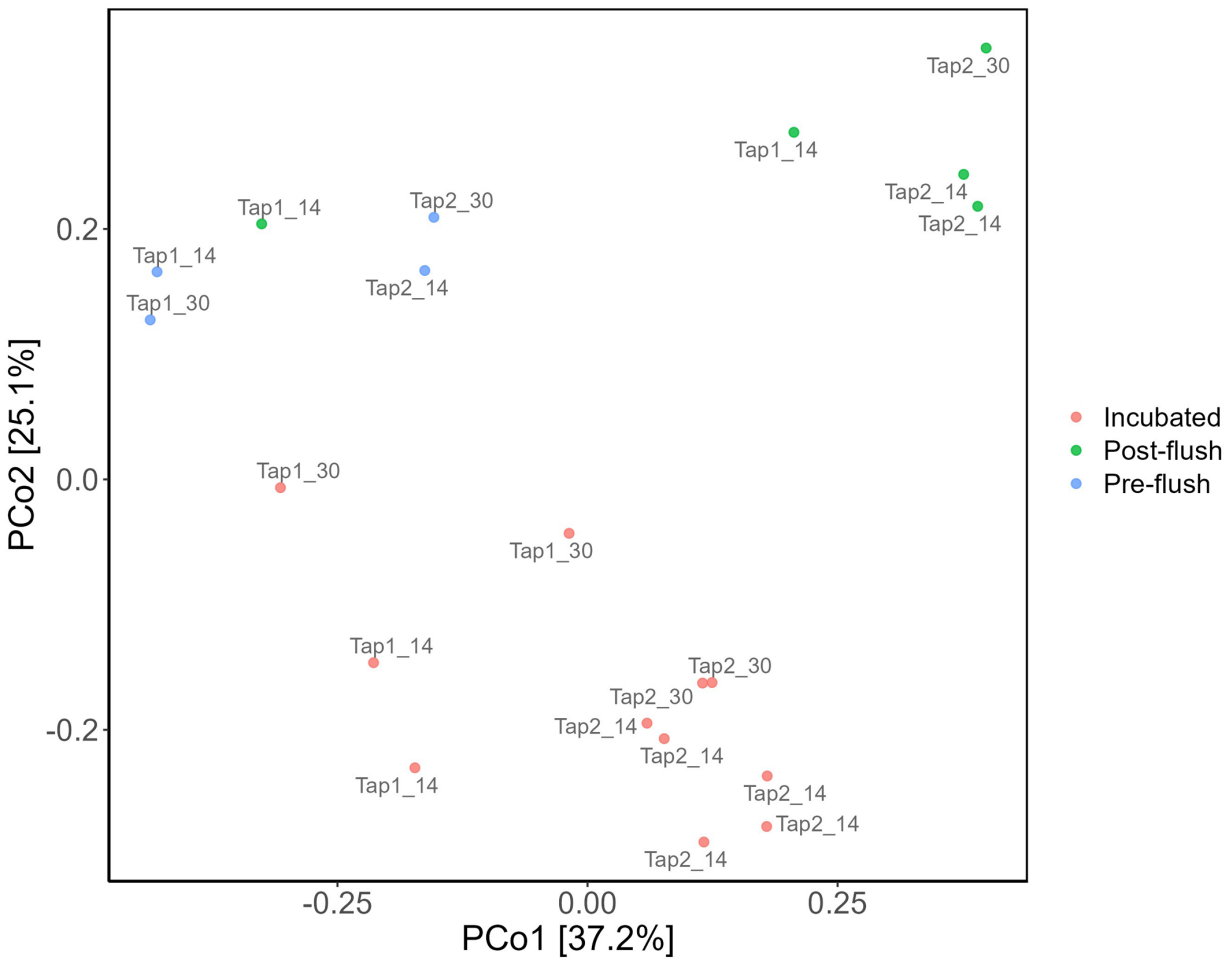
Bacterial community dissimilarity based on sample groups. Principal coordinate analysis (PCoA) of amplicon data based on Bray–Curtis dissimilarity.

To elucidate if increasing cell density could be a feasible alternative to improve DNA yield before performing downstream molecular analysis of low-biomass RO tap water, bacterial community profile from a controlled stagnation experiment was examined. One contaminant genus was detected to be highly abundant, i.e., *Allorhizobium, Neorhizobium, Pararhizobium*, and *Rhizobium* from Proteobacteria phylum (66%–94% of the relative abundances in controls; Figure 6A), most likely cross-contamination during the workflow or from the sequencing platform. Six more OTUs present in high abundance in the controls (Figure 6A; also confirmed by the least *p-*values based on DESeq analysis, data not shown) but not in samples were removed to create the second heatmap (Figure 6B) and ordination plot (Figure 7). The ordination plot clearly shows distinct bacterial communities of incubated samples compared to the pre-, and post-flush while the heatmap reveals the top 20 most abundant genera present in RO tap water from each sample group after removal of taxa that are highly enriched in the controls (Figure 6B).

Further analysis concerning the difference clustering of the bacterial communities (Figure 7) observed was done with multiple pairwise comparisons, revealing significant differences for all sample group pairs, except for pre- vs. post-flush samples. The tap location was found to have a significant effect on the bacterial community composition (PERMANOVA, *p* = 0.01) which can be attributable to the compositional variance in the bacterial community between both taps: samples from tap 1 have more consistent communities compared to tap 2 (Betadisper, *p* = 0.25, Supplementary Figure S4). Nevertheless, the distinction in the bacterial community between three sample groups (incubated, pre- and post-flush) can further be explained by 17 OTUs (out of 139 total OTUs identified from all samples) that made up the core members (cut-off relative abundance of at least 0.0001; and present in at least 50% of samples; Supplementary Figure S3). Notably, two genera were detected in all samples (100% of samples), namely *Porphyrobacter*, the most abundant genus, and *Blastomonas* (Figure 7B; Supplementary Figure S3). Both bacterial genera belong to the family Sphingomonadaceae which could serve as a strong potential indicator bacteria for RO tap water microbial water quality. Collectively, these findings highlight that increasing the number of samples (i.e., one-liter volume from multiple taps) allows identification of the resident bacterial community in RO tap bulk water, together with using the correct filter (PC 0.2 μm) and optimized DNA extraction protocol as well as incorporating multiple controls. Additionally, increasing cell density via incubation can be an easy and effective strategy to enhance DNA yield, allowing accurate identification of the core microbiome of low-biomass RO tap water. Incubation of RO tap water implies the thriving bacterial groups in RO water able to consume (low-amount) naturally assimilable organic carbon present.

## 4 Discussion

### 4.1 Filter membrane pore size has no linear correlation with extracted DNA yield

We hypothesized that biomass filtration efficiency will correlate with recovered DNA extracts; the higher efficiency will result in higher DNA yields. However, our results showed no linear correlation between DNA yield and pore size filter (biomass retention efficiency; Figure 3). A smaller pore size filter did not equate to higher DNA yield which can be explained by premature clogging, facilitating DNA degradation (Abreu-Silva et al., 2023). A previous study has shown that a larger pore size filter favored long (environmental) DNA fragment retention compared to short DNA (i.e., less degraded DNA; Jo et al., 2020) which likely contributes to high yields of DNA recovery.

This study identifies that a PC filter membrane with 0.2 μm pore size enables high recovery of DNA extract from low-biomass RO tap water. This filter remarkably outperforms the other four filter membranes tested, in agreement with similar previous studies that reported high DNA yield and 16S gene abundance of low microbial load water samples extracted with a PC filter (Minamoto et al., 2016; Abreu-Silva et al., 2023). PC filter membrane has a straight-through pore-channel structure, less surface roughness, least hydrophilic (higher surface hydrophobicity), and highest wettability (Dizge et al., 2011; Abreu-Silva et al., 2023) that may explain higher DNA yields and low background contaminant (16S rRNA copy number) obtained. PC filter with 0.2 μm is known to retain less protein and carbohydrate (Dizge et al., 2011), which can interfere with DNA extraction and severely affect DNA purity (Abdel-Latif and Osman, 2017). Aggregates of the solubilized microbial products, including its DNA, have been reported to be greater for PC membranes than other membranes with interconnected pore geometry (e.g., PES and MEC; Dizge et al., 2011).

However, it should be noted that intra- and intervariations between filter material and the nested pore size exist. Filter pore sizes are known to be different in their nominal value described by the manufacturer, the size of any pore can widely vary (Deiner et al., 2018). That being said, the pore size of a filter membrane is rather a probability distribution and not an exact (homogenous). Investigation on the micrograph of the pore structures of filter membranes used was out of the scope of the current study and the pore size value itself was not determined analytically, but for PC filter specifically, the scanning electron microscopy images are available elsewhere (Tung and Chuang, 2002; Paoli et al., 2020). Our results show that the PC filter gives the best DNA yield and low background 16S rRNA gene copies but the manufacturer has stated that the water volume filter should be a maximum of two liters. A composite sampling, i.e., splitting biomass filtration into multiple filters could be considered if larger sampling volumes are needed and how it affects the DNA concentration and microbial community remains to be investigated in future studies. Future works should also explore how different pore sizes that are dependent on filter materials interact with (extracellular) DNA could explain different DNA yields. Nevertheless, our results emphasize that using a PC filter with 0.2 μm pore size and multiple smaller volumes is reasonable and provides a good representation (i.e., rarefaction curves show that most bacterial taxa were captured, Supplementary Figure S5) of the bacterial community composition in low-biomass RO tap water.

### 4.2 Differences in bacterial diversity following incubation to enhance DNA yield

First, in terms of absolute abundance (i.e., number of different OTUs), the low number of OTUs (139 OTUs) detected reflects the low bacterial cell numbers in the bulk water, as found elsewhere (Kantor et al., 2019). Increasing sampling volume does not fundamentally address the problem (Stamps et al., 2018; Putri et al., 2021) due to the sparse distribution of bacterial cells in RO bulk water (i.e., low cell density, low ATP, and 16S gene copy number, this study). The tap water incubation procedure increased bacterial cell density which correlated with an increase in DNA yield and gene copy number (Figure 5B). However, the gene copy number had less variability compared to the DNA yield (Figures 5A,B), and thus more indicative of the high cell density. Adding 16S rRNA gene copies parameter allows for reducing false negative of low DNA yield samples before proceeding with amplicon sequencing. Second, in terms of beta diversity analysis we observed differences in bacterial communities in terms of overall richness (i.e., number of OTUs; Figure 6B) after deducting from the contaminant detectable in the controls (Figure 6B). The contaminant genus is known to be isolated from wastewater samples (Bigott et al., 2022) and appeared exceptionally high (~66%–94%) in the controls, likely to be cross-contamination from the sequencer (and/or sequencing provider). The present study also rules out more contaminant genera: *Ralstonia, Sphingomonas, Corynebacterium_1, Methylobacterium, Idiomarina*, and *Rhodococcus* that were present in high abundant and high coverages in the control and samples irrespective of cell density regimes. All these genera have been previously reported as common contaminant taxa from low-biomass environments (Salter et al., 2014).

Post-flush network samples had the highest diversity, i.e., more appearance of low abundance OTUs compared to incubated and pre-flush samples (Figure 6B). This difference could reflect the actual environmental heterogeneity between those samples, such as temperature, sample confinement (e.g., materials) during incubation, and hydrodynamics conditions (Neu et al., 2018; Proctor et al., 2018; Dai et al., 2020). Nonetheless, the core bacterial community (Supplementary Figure S3) between post-flush (low DNA yield) and incubated tap water samples (high DNA yield) from the same environment converged, showing that the incubation strategy works in reflecting the true diversity of low-biomass chlorinated RO drinking water samples. It is well acknowledged that amplicon sequencing cannot exhaustively reveal the total microbial content in a given sample and can only serve as a tool to characterize the dominant taxa in a sample and for comparative assessment between different samples or grouping factors. However, it remains a faster and more cost-effective method for identifying microbial taxa compared to shotgun metagenomics. Performing quantitative PCR targeting a specific bacterium (other than the total 16S rRNA gene copies) that may be predominant in the sample microbiome composition can also be done to ensure that a low DNA yield sample has higher biological signals than the control (Zhang et al., 2015).

Based on the core microbiome (i.e., shared bacterial taxa) analysis using cutoff: taxon presence in at least 50% of samples (occupancy-based) and a total abundance higher than 0.001% across all samples (Custer Gordon et al., 2023), we identified two genera that were found consistently throughout all cell density regimes, either pre-flush (*in-situ* stagnation), post-flush or incubated samples: *Porphyrobacter* and *Blastomonas*, highlighting their importance in the (chlorinated) RO-produced drinking water biological stability*. Porphyrobacter* has been reported to proliferate after surviving chlorine dioxide disinfection (Ma et al., 2020) while *Blastomonas* is a dominant bacterial genus found in tap water resistant to chlorine dioxide (Paduano et al., 2020; Sala-Comorera et al., 2020) and biofilm in a chlorinated distribution system (Buffet-Bataillon et al., 2010). These two genera are very likely to survive the residual chlorine present in the RO bulk water, explaining their high abundance. The overall bacterial community profile from this study could serve as baseline data for chlorinated RO-produced drinking water. The genus level information provided also highlights potential ecological attributes of those two genera that are often overlooked when using a higher level of taxonomical information (e.g., phylum, class; Hermans et al., 2017). We did the comparison of bacterial community from the bulk water previously at the upstream part of KAUST RO tap water before and after chlorination point (Farhat et al., 2022) and found a high abundant of *Blastomonas* genus in concurrence with the current study, showing that this bacterium is the actual resident within chlorinated RO tap network that is resistant to chlorine. Further studies could be done to investigate temporal (and spatial) variations (e.g., physicochemical conditions, sampling from different buildings) from chlorinated RO tap water and how these two genera correlates with those factors.

In addition, 17 OTUs (Supplementary Figure S3) were identified successfully as core microbiomes of RO bulk water that are highly adapted to oligotrophic (low level of AOC) conditions and chlorine disinfection specific to our drinking water network. It is worth noting, that some taxa were enriched in pre-flush but not post-flush or incubated samples (Figure 6B) such as *Dechloromonas*, *Novosphingobium*, and *Piscinibacter*. *Dechloromonas* has been reported to dominate the biofilm in disinfected drinking water, playing a role in inhibiting corrosion (Wang et al., 2017a). *Novosphingobium* has been known to increase its antibiotic resistance gene expression via biotransformation with antibiotic (Ciprofloxacin)-chlorination products (Wang et al., 2017b). Meanwhile, *Piscinibacter* has been reported to predominate cultured biofilm growing in chlorinated polyvinyl chloride that can utilize a single carbon compound (van der Kooij et al., 2017). We hypothesized that these bacteria can grow in pre-flush samples following the rise in assimilable organic carbon (AOC) due to the stagnation and depletion of residual chlorine. The range of AOC in pre-flush samples was between 0.025–0.05 μg (1 μg AOC = 10^6^ cells/mL; Hammes and Egli, 2005) AOC after stagnation.

In summary, this study shows that a strategy employing a good filter (PC 0.2 μm) membrane for (microbial) DNA extraction with low background contaminant, optimizing DNA extraction protocol, incorporating multiple controls, having multiple smaller (1 L) sampling volumes, and increasing cell density via incubation allows to profile microorganisms in low-biomass RO tap water conclusively. Future studies should investigate how different filter membrane materials and pore sizes affect different microbial endpoints (eukaryotes, fungi, viruses) in this particular type of water. Different groups of microorganisms present in low-biomass tap water will probably have different cut-offs of filter membranes (type/pore size). We also observed a shift in abundance of the core microbiome between pre-, post-flush, and incubated samples that warrant further investigation of how these communities change across seasonal and spatial variations. Amplicon sequencing analysis also does not delineate active and dead cells so further examination could be performed, such as using propidium monoazide-quantitative polymerase chain reaction (PMA-qPCR) targeting *Porphyrobacter* and/or *Blastomonas*.

### 4.3 Practical implications

There are practical implications to the findings of this study. First, circumventing the low DNA yield from low-biomass RO tap water before performing downstream DNA-based analysis can be done with multiple smaller (i.e., one-liter) sampling volumes and incubation of the bulk water to reach higher bacterial cell density (with naturally occurring AOC). These two approaches allow for conclusively profiling the bacteria present and influence the biological stability in the premise plumbing and the distribution network conditions. Our current results expand prior work of the optimized DNA extraction protocol (i.e., DNEasy Power Water kit) that was adopted in this study for low-range bacterial cell concentration (10^3–^10^5^ cells/mL; Vosloo et al., 2019). Importantly, to the authors’ knowledge, our study is the first of its kind to identify the best filter membrane (i.e., PC 0.2 μm) for DNA extraction of (chlorinated) low-biomass RO-produced drinking water with the highest DNA yield and lowest background copy number of 16S rRNA gene. Second, incorporating low-biomass blanks is crucial as well as subjecting it to the same amplicon sequencing/bioinformatics pipeline for the best results’ interpretation of microbial community analysis from this type of water. Here, we added a Milli-Q and sterilized (autoclaved) version of incubated RO tap water to the blanks/controls filtered through the same filter membrane in addition to the well-established blanks (no template DNA blank, and unused virgin/sterile filter membrane blank) that are commonly used, and thus we also recommend future studies to incorporate this type of blanks.

## 5 Conclusion

A high quantity of (microbial) DNA yield is the most important attribute before performing DNA-based analysis like amplicon sequencing because DNA purification starts from the highest yields possible. The present study comprehensively investigated the impact of (i) different filter membrane types (materials and their pore sizes); (ii) different cell density regimes and (iii) the impact of increasing DNA yield via incubation of the bulk water with no nutrient spiking affecting a set of outcome parameters (DNA yield, 16S rRNA gene copy number, and bacterial community composition) focusing on low-biomass (chlorinated) RO-produced tap water. The following conclusions were drawn:

- After examining five different filter membranes (mixed ester cellulose 0.2 μm, polycarbonate 0.2 μm, polyethersulfone 0.2 and 0.1 μm, polyvinylidene fluoride 0.1 μm), we showed that smaller membrane pore size solely did not increase the DNA yield of low-biomass RO-produced tap water. DNA yield is proportional to cell density and substantially dependent on filter membrane extraction efficiency (i.e., membrane material and its pore size).
- A polycarbonate filter membrane with 0.2 μm markedly outperformed in terms of quantity (highest DNA concentration/yield) and quality (lowest background 16S gene copy number).
- Incubation (i.e., increased cell density) of RO-produced tap water enhances the obtained DNA yield and identifies the core microbiome accurately with a one-liter sampling volume and an increased number of samples.
- We successfully identified the two bacterial genera: *Porphyrobacter* and *Blastomonas* that were present in high abundance and persist in the chlorinated RO tap water network samples and how local conditions/processes (e.g., plumbing materials, hydrodynamics regime, and daily stagnation) may cause a shift and emergence of completely different dominant bacterial taxa.
- Incorporating multiple blanks is crucial to eliminate contaminant taxa for performing amplicon sequencing of low-biomass tap water.

Collectively, our study provides the first experimental evidence to improve the reliability of microbial community analysis with a cost-effective strategy (i.e., incubation of bulk water via naturally assimilable organic carbon) to enhance DNA yield. Eventually, the current study expands the alternative method to help reach a more standardized study of drinking water microbiology in high-quality (ultra-pure) water produced by membrane treatment like RO.

## Data availability statement

All relevant data are within the article/Supplementary material. Further inquiries can be directed to the corresponding author.

## Author contributions

RP: Writing – review & editing, Writing – original draft, Software, Methodology, Investigation, Formal analysis, Data curation, Conceptualization. JV: Writing – review & editing, Supervision, Resources, Project administration, Funding acquisition, Conceptualization. NF: Writing – review & editing, Supervision, Project administration, Methodology, Formal analysis, Data curation, Conceptualization.
