# Supplementary material for: Enhancing the DNA yield intended for microbial sequencing from a low-biomass chlorinated drinking water
Source: Front Microbiol. 2024 May 24;15:1339844. doi: 10.3389/fmicb.2024.1339844 (PMC11157071; doi:10.3389/fmicb.2024.1339844)
Supplement: Supplementary file 1 [file Data_Sheet_1.docx]

Supplementary Material

Enhancing the DNA yield intended for microbial sequencing from a low-biomass chlorinated drinking water

Ratna E. Putri^1^, Johannes S. Vrouwenvelder^1,2^, Nadia Farhat^1*^

^1^Water Desalination and Reuse Center, Biological and Environmental Science and Engineering Division, King Abdullah University of Science and Technology, Thuwal 23955-6900, Saudi Arabia

^2^Department of Biotechnology, Faculty of Applied Sciences, Delft University of Technology, Van der Maasweg 9, HZ Delft 2629, The Netherlands

*** Correspondence:**Corresponding Author
nadia.farhat@kaust.edu.sa


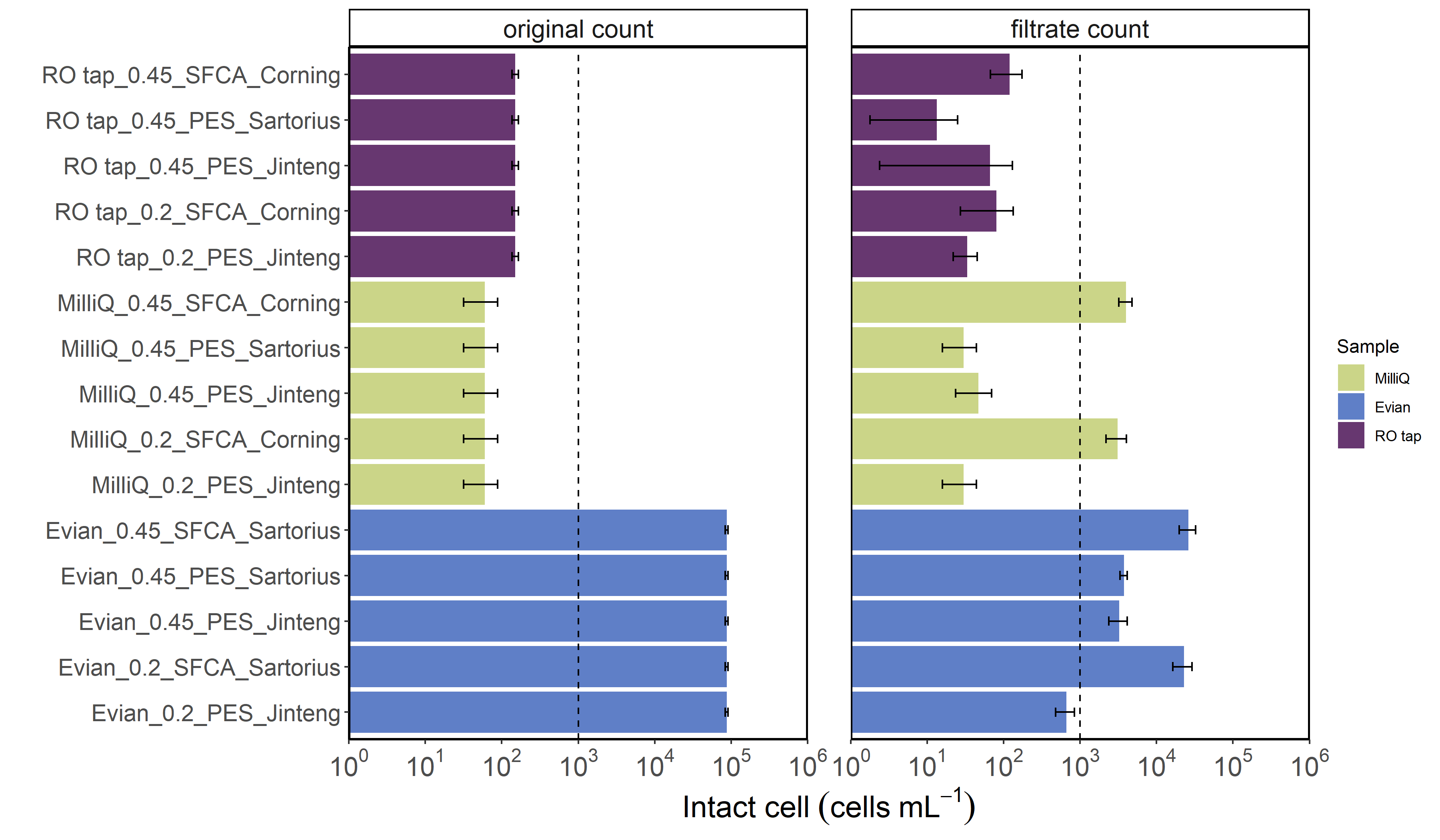


**Supplementary Figure S1. Intact bacterial cell count in the original and filtrate passing through different commercial syringe membrane filters for different water samples (RO tap water, MilliQ, Evian bottled water).** The y-axis label contains the following convention ‘water type_membrane syringe filter pore size_ membrane syringe filter type_brand/manufacturer’. The dashed vertical line set on the 10^3^ cells/mL is the method detection limit of the flow cytometry used in this study. Among different pore sizes tested, SFCA (Surfactant-free cellulose acetate) filtrates overestimate the count of intact cell compared to the PES (polyethersulfone) that had the same pore size.

**
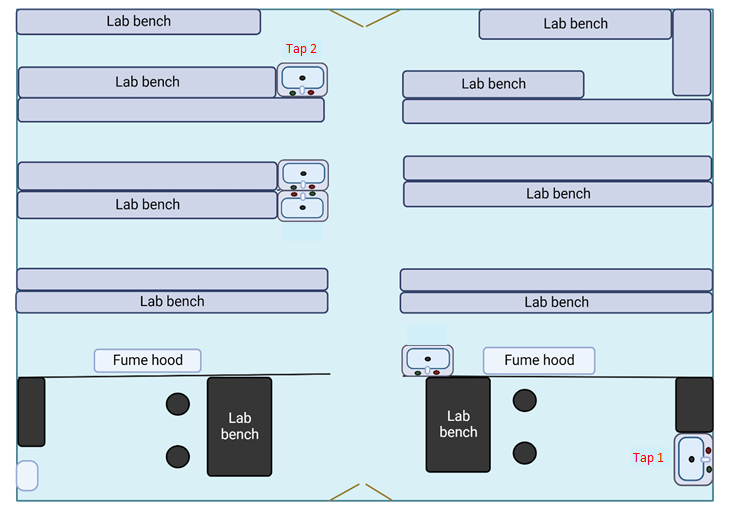
**

**Supplementary Figure S2. Schematic of tap sampling location used in this study.** Premised taps are located in the Water Desalination and Reuse Center laboratory, KAUST, Kingdom of Saudi Arabia. All samples were collected from cold water taps only.


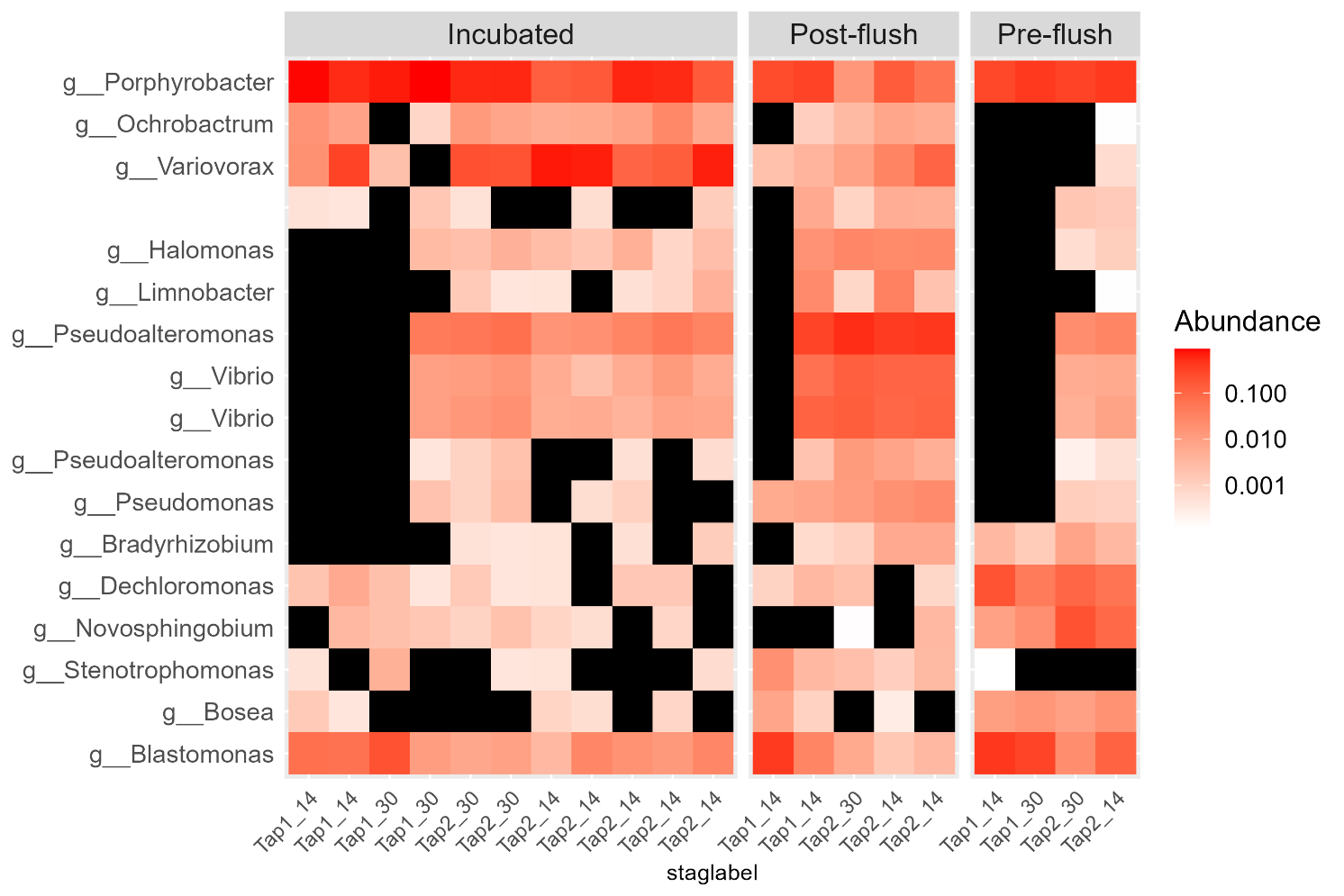


**Supplementary Figure S3. Core genera identified in RO bulk water irrespective of sample groups (controlled stagnation experiment).** Samples were pruned from the seven most abundant taxa present in controls. Only OTU present in at least two samples, with more than 50% prevalence and relative abundance of at least 0.0001 were defined as core members. The x-axis is labeled based on the tap location and stagnation period (individual sample is shown). On the y-axis is the genus (where available) assigned to the OTUs; a row with no genus name indicates the OTU(s) assignment to the higher taxonomic ranks but not to the genus level. Samples are grouped by cell density regimes: incubated (post-flush, dechlorinated), post-flush, and pre-flush sample.

**
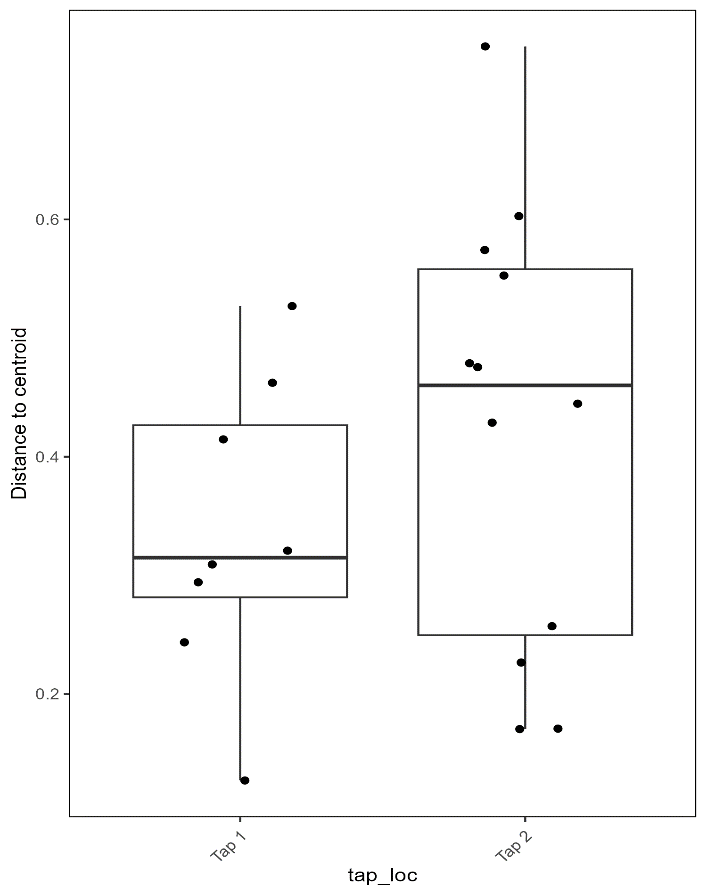

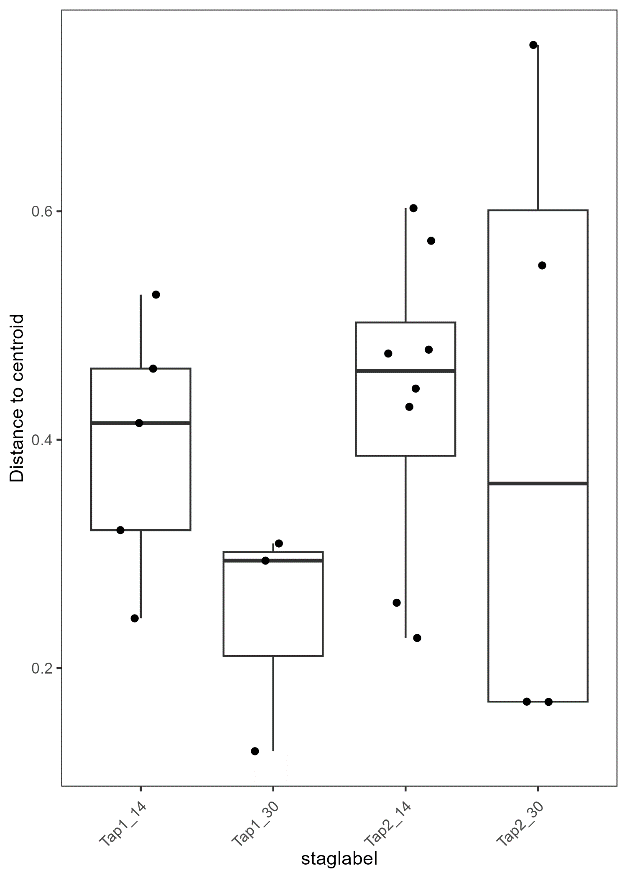
**

**A**

**B**

**Supplementary Figure S4. Betadisper analysis boxplot of stagnation experiment samples.** The lower value of distance to the centroid (y-axis) means that the sample within that group/category had more consistent bacterial communities. Betadisper *P* value = 0.25 meaning that heterogeneous group dispersions of samples within different taps are not significant. Samples were stratified based on (A) tap location, and (B) stagnation periods within each tap. Between different taps, samples from Tap 1 had the most consistent communities compared to Tap 2, even though variation between different stagnation periods were observed within each tap.

**Supplementary Figure S5. Rarefaction curves of post-flush samples depict the sequencing depth effort.** Samples coded with D66 (Tap1), D68 (Tap 1), D71 (Tap 2), D77 (Tap 2), and D82 (Tap 2) are all post-flush samples from the controlled stagnation experiment (please refer to S2 Fig in regards to tap location).


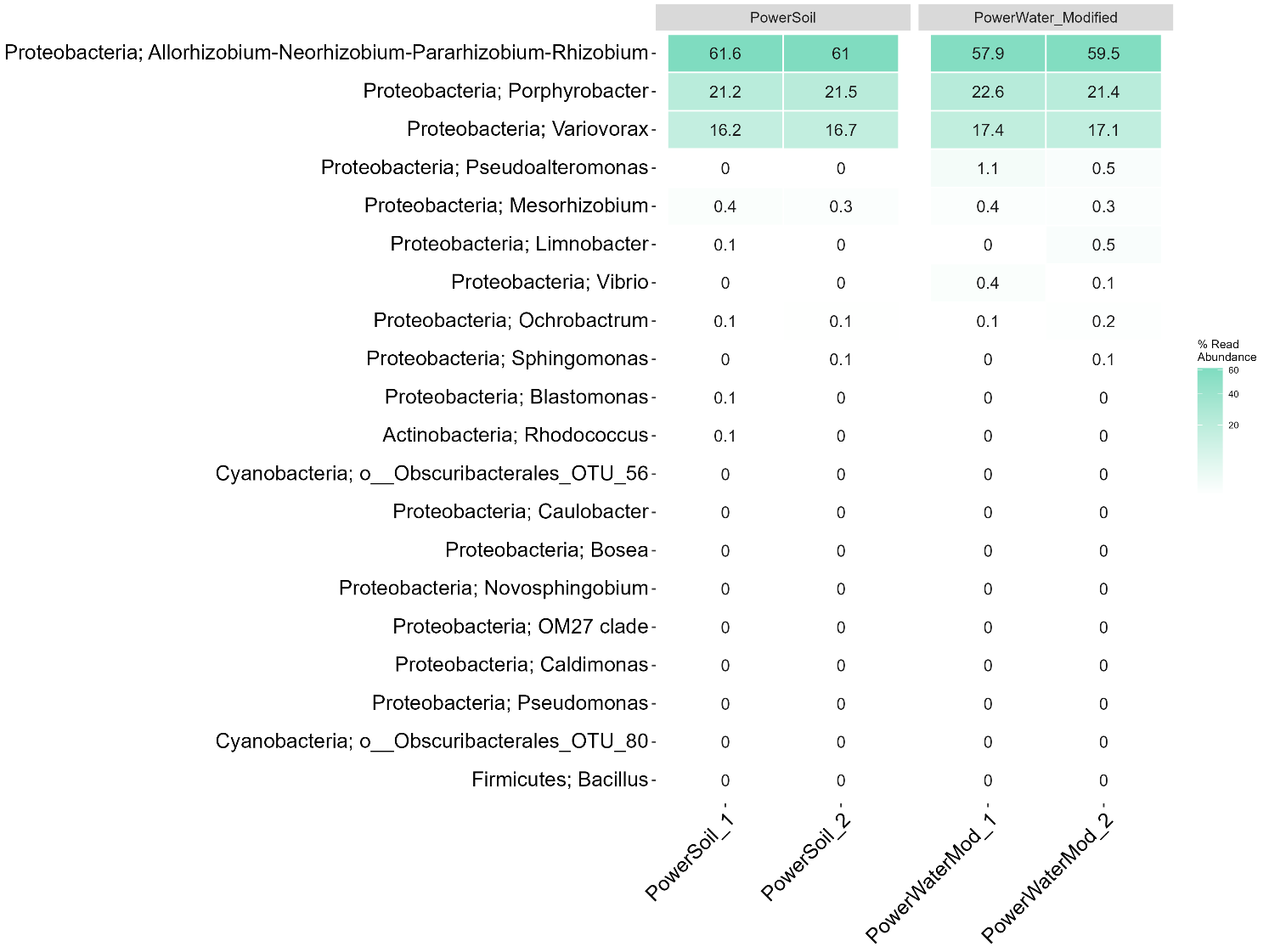


**Supplementary Figure S6. Heatmaps of 1 L incubated dechlorinated RO tap water extracted with different kits (PowerWater Modified VS PowerSoil).** Each column represents the relative abundance from two independent replicate samples and each row shows the 20 most abundant OTUs across the samples.

**Supplementary Table S1. Mean passage percentage (± SD, n=3) of filtrate water bacterial communities through different commercial syringe filter materials and pore sizes.** Different syringe filters used are surfactant-free cellulose acetate (SFCA) from three manufacturers, and polyethersulfone from three manufacturers with different pore sizes: 0.22 µm (PES-Jinteng, SFCA-Sartorius/Corning); 0.45 µm (PES-Jinteng, PES-Sartorius, SFCA-Corning).

| **source** | **intact cell count in original water (cells/mL)** | **filter pore size and material** | | | | |
| --- | --- | --- | --- | --- | --- | --- |
|  |  | **0.45 µm** | | | **0.22 µm** | |
|  |  | **SFCA** | **PES -J** | **PES-S** | **SFCA** | **PES-J** |
| MilliQ | 60 ± 28 | 7850 ± 5020.45% | 62.5% ± 17.67% | 50 ± 0% | 5637 ± 4331.02% | 62.5 ± 53.03% |
| RO tap | 150 ± 14 | 100 ± 0% | 19.5 ± 7.77% | 13.5 ± 0.70% | 44.5 ± 43.13% | 19.5 ± 7.77% |
| Evian | 87566 ± 3475 | 29.82 ± 7.20% | 3.73 ± 1.07% | 4.26 ± 0.43% | 25.87 ± 6.57% | 0.75 ± 0.23% |

**Supplementary Table S2. Summary statistics of samples DNA yield extracted with various filter membranes.** N is the number of samples within each category/group. SD is the standard deviation, SE is the standard error and CI is the confidence interval. DNA yield is calculated from the total DNA obtained in 50 µL of final elution volume. NA means not available (i.e. not calculated/not enough replicates).

| **sample_type** | **group** | **membrane_type** | **variable** | **N (sample number)** | **value** | **SD** | **SE** | **CI** |
| --- | --- | --- | --- | --- | --- | --- | --- | --- |
| MilliQ | Control | MEC | dna_yield | 1 | 0.5 | NA | NA | NA |
| RO tap sterile | Control | MEC | dna_yield | 1 | 0.5 | NA | NA | NA |
| Virgin membrane | Control | MEC | dna_yield | 1 | 0.6 | NA | NA | NA |
| MilliQ | Control | PC | dna_yield | 1 | 1.0 | NA | NA | NA |
| RO tap sterile | Control | PC | dna_yield | 1 | 0.5 | NA | NA | NA |
| Virgin membrane | Control | PC | dna_yield | 1 | 0.5 | NA | NA | NA |
| MilliQ | Control | PES | dna_yield | 1 | 0.5 | NA | NA | NA |
| RO tap sterile | Control | PES | dna_yield | 1 | 0.5 | NA | NA | NA |
| Virgin membrane | Control | PES | dna_yield | 1 | 0.5 | NA | NA | NA |
| MilliQ | Control | PVDF | dna_yield | 1 | 0.5 | NA | NA | NA |
| RO tap sterile | Control | PVDF | dna_yield | 1 | 0.5 | NA | NA | NA |
| Virgin membrane | Control | PVDF | dna_yield | 1 | 0.5 | NA | NA | NA |
| Evian | Evian | MEC | dna_yield | 2 | 1.6 | 0.4 | 0.3 | 3.2 |
| Evian | Evian | PC | dna_yield | 2 | 140.5 | 4.9 | 3.5 | 44.5 |
| Evian | Evian | PES | dna_yield | 2 | 20.1 | 3.4 | 2.4 | 30.5 |
| Evian | Evian | PVDF | dna_yield | 2 | 0.6 | 0.2 | 0.1 | 1.6 |
| RO tap | Incubated | MEC | dna_yield | 4 | 24.4 | 15.3 | 7.7 | 24.4 |
| RO tap | Incubated | PC | dna_yield | 8 | 49.0 | 46.2 | 16.3 | 38.6 |
| RO tap | Incubated | PES | dna_yield | 3 | 9.4 | 1.9 | 1.1 | 4.7 |
| RO tap | Incubated | PES Sterivex | dna_yield | 4 | 7.2 | 5.7 | 2.9 | 9.1 |
| RO tap | Incubated | PVDF | dna_yield | 4 | 4.0 | 3.2 | 1.6 | 5.1 |
| RO tap | Post-flush | MEC | dna_yield | 2 | 0.5 | 0.0 | 0.0 | 0.0 |
| RO tap | Post-flush | PC | dna_yield | 2 | 0.5 | 0.0 | 0.0 | 0.0 |
| RO tap | Post-flush | PES | dna_yield | 2 | 0.5 | 0.0 | 0.0 | 0.0 |
| RO tap | Post-flush | PVDF | dna_yield | 2 | 0.5 | 0.0 | 0.0 | 0.0 |
| RO tap | Preflush | MEC | dna_yield | 2 | 1.0 | 0.7 | 0.5 | 6.4 |
| RO tap | Preflush | PC | dna_yield | 4 | 13.9 | 26.8 | 13.4 | 42.6 |
| RO tap | Preflush | PES | dna_yield | 2 | 0.5 | 0.0 | 0.0 | 0.0 |
| RO tap | Preflush | PES Sterivex | dna_yield | 2 | 0.68 | 0.25 | 0.18 | 2.22 |
| RO tap | Preflush | PVDF | dna_yield | 2 | 0.5 | 0 | 0 | 0 |

**Supplementary Table S3A. Fold increase in total DNA per filtration volume after using the modified DNEasy PowerWater extraction protocol**

| **Sample name** | **Total cell concentration (cells/mL)** | **DNA concentration (ng/µl)** | **Total DNA per total filtration volume (ng/L)** | **Fold increase** |
| --- | --- | --- | --- | --- |
| Evian - modified | **5.18E+04** | 0.06 | 2.3 | 5.8 |
| Evian - unmodified | **5.18E+04** | 0.01 | 0.4 |  |

**Supplementary Table S3B. Increased DNA yield obtained from Evian sample with two different extraction protocol**

| **Sample** | **Kit** | **replicate** | **DNA concentration (ng/µL)** | **Average (± SD)** | **Fold difference** |
| --- | --- | --- | --- | --- | --- |
| Evian | DNEasy Power Water Modified | 1 | 1.52 | **1.77 ± 0.35** | 9.6 |
|  |  | 2 | 2.02 |  |  |
|  | DNA PowerSoil | 1 | 0.24 | **0.18 ± 0.07** |  |
|  |  | 2 | 0.13 |  |  |

**Supplementary Table S4. Original and filtrate intact cell count from syringe filter experiment.** PES syringe filter with varied sizes were used. The detection limit of the flow cytometry used to measure the bacterial cell count is 1000 cells/mL.

| Sources | Original count (Cells/mL) | Filtrate count with 0.45 µm pore size (Cells/mL) | Filtrate count with 0.2 µm pore size (Cells/mL) | Filtrate count with 0.1 µm pore size (Cells/mL) |
| --- | --- | --- | --- | --- |
| Evian | 62040 | 9560 | 9060 | 1900 |
| Evian | 61300 | 8580 | 7560 | 720 |
| Evian | 62200 | 8700 | 7160 | 500 |
| RO tap | 220 | 120 | 120 | 20 |
| RO tap | 220 | 180 | 100 | 20 |
| RO tap | 1820 | 1260 | 1580 | 540 |
| RO tap | 3100 | 1180 | 2420 | 760 |

**Supplementary Table S5A. Significance values of factors affecting DNA yield based on ANOVA.** *P* value < 0.05 is considered significant.

|  | ***P* value** |
| --- | --- |
| **Factors** | **DNA yield** |
| Sample group (cell density) | **0.01398** |
| Filter membrane type | **0.004543** |

**Supplementary Table S5B. Significance values, differences between means, and confidence intervals of DNA yield ANOVA model as a function of different filter membranes on post-hoc TukeyHSD (Tukey Honest Significant Differences) test.** *P* value < 0.05 is considered significant (denoted by numbers in bold with a *).

|  | **Parameter: DNA yield** | | | |
| --- | --- | --- | --- | --- |
| **Comparison** | **difference between means** | **lower CI (5%)** | **upper CI (95%)** | ***p* adj** |
| **PC-MEC*** | 29.38 | 7.87 | 50.89 | **0.003*** |
| PES Sterivex-MEC | -6.29 | -35.78 | 23.19 | 0.97 |
| PES-MEC | -1.30 | -25.22 | 22.62 | 0.99 |
| PVDF-MEC | -6.52 | -29.95 | 16.91 | 0.93 |
| **PES Sterivex-PC*** | -35.68 | -63.65 | -7.69 | **0.006*** |
| **PES-PC*** | -30.68 | -52.71 | -8.65 | **0.002*** |
| **PVDF-PC*** | -35.90 | -57.41 | -14.39 | **0*** |
| PES-PES Sterivex | 4.99 | -24.88 | 34.86 | 0.99 |
| PVDF-PES Sterivex | -0.22 | -29.71 | 29.26 | 0.99 |
| PVDF-PES | -5.22 | -29.14 | 18.70 | 0.98 |

Supplementary Table S5C. Significance values, differences between means, and confidence intervals of 16S gene copy numbers ANOVA model as a function of different filter membranes on post-hoc Tukey HSD (Tukey Honest Significant Differences) test. *P* value < 0.05 is considered significant (denoted by numbers in bold with a *).

|  | **Parameter: 16S rRNA gene copies** | | | |
| --- | --- | --- | --- | --- |
| **comparison** | **difference between means** | **lower CI (5%)** | **upper CI (95%)** | ***p* adj** |
| **PC-MEC** | 273903 | 223804 | 324003 | **0*** |
| PES-MEC | -252 | -52797 | 52292 | 0.99 |
| PVDF-MEC | -38235 | -88334 | 11865 | 0.16 |
| **PES-PC** | -274156 | -326700 | -221611 | **0*** |
| **PVDF-PC** | -312138 | -362237 | -262038 | **0*** |
| PVDF-PES | -37982 | -90527 | 14563 | 0.19 |

**Supplementary Table S5D. Summary statistics of samples for total bacterial (16S rRNA) gene abundance from each group extracted with various filter membranes.** N is the number of samples within each category/group. SD is the standard deviation, SE is the standard error and CI is the confidence interval. Cp_num is 16S gene copy numbers. NA means not available (i.e. not calculated/not enough replicates).

| **sample_type** | **group** | **mem_type** | **mem_size** | **variable** | **N** | **value** | **sd** | **se** | **ci** |
| --- | --- | --- | --- | --- | --- | --- | --- | --- | --- |
| MilliQ | Control | MEC | 0.2 µm | cp_num | 1 | 1.00E+03 | NA | NA | NA |
| RO tap sterile | Control | MEC | 0.2 µm | cp_num | 1 | 1.00E+03 | NA | NA | NA |
| Virgin membrane | Control | MEC | 0.2 µm | cp_num | 1 | 1.43E+03 | NA | NA | NA |
| MilliQ | Control | PC | 0.2 µm | cp_num | 1 | 1.00E+03 | NA | NA | NA |
| RO tap sterile | Control | PC | 0.2 µm | cp_num | 1 | 1.00E+03 | NA | NA | NA |
| Virgin membrane | Control | PC | 0.2 µm | cp_num | 1 | 1.00E+03 | NA | NA | NA |
| MilliQ | Control | PES | 0.1 µm | cp_num | 1 | 1.00E+03 | NA | NA | NA |
| RO tap sterile | Control | PES | 0.1 µm | cp_num | 1 | 1.00E+03 | NA | NA | NA |
| Virgin membrane | Control | PES | 0.1 µm | cp_num | 1 | 2.95E+03 | NA | NA | NA |
| MilliQ | Control | PVDF | 0.1 µm | cp_num | 1 | 1.00E+03 | NA | NA | NA |
| RO tap sterile | Control | PVDF | 0.1 µm | cp_num | 1 | 1.00E+03 | NA | NA | NA |
| Virgin membrane | Control | PVDF | 0.1 µm | cp_num | 1 | 1.00E+03 | NA | NA | NA |
| Evian | Evian | MEC | 0.2 µm | cp_num | 1 | 1.00E+03 | NA | NA | NA |
| Evian | Evian | PC | 0.2 µm | cp_num | 1 | 1.50E+06 | NA | NA | NA |
| Evian | Evian | PES | 0.1 µm | cp_num | 1 | 8.90E+04 | NA | NA | NA |
| Evian | Evian | PVDF | 0.1 µm | cp_num | 1 | 1.00E+03 | NA | NA | NA |
| RO tap | Incubated | MEC | 0.2 µm | cp_num | 2 | 1.17E+05 | 9.35E+04 | 6.61E+04 | 8.40E+05 |
| RO tap | Incubated | PC | 0.2 µm | cp_num | 2 | 1.90E+05 | 1.98E+04 | 1.40E+04 | 1.78E+05 |
| RO tap | Incubated | PES | 0.1 µm | cp_num | 1 | 1.02E+05 | NA | NA | NA |
| RO tap | Incubated | PVDF | 0.1 µm | cp_num | 2 | 2.91E+03 | 7.74E+02 | 5.48E+02 | 6.96E+03 |

**Supplementary Table S6A. Average of quantitative parameters (±SD) consisting of total/intact cell concentration, 16S rRNA gene copy numbers, DNA yield, and ATP concentration from a controlled stagnation experiment.** The sample volume processed for this part of the experiment was one liter and each group was done in duplicate except for pre-flush samples that have only one occurrence in each group. N.d. means not detected.

|  |  | **Controlled stagnation: 30 days** | | | |  | **Controlled stagnation: 14 days** | | | |  |
| --- | --- | --- | --- | --- | --- | --- | --- | --- | --- | --- | --- |
|  |  | **TCC(cells/mL)** | **ICC(cells/mL)** | **DNA yield (ng)** | **ATP (pg/ml)** | **copies of 16S gene/µL** | **TCC(cells/ml)** | **ICC(cells/mL)** | **DNA yield (ng)** | **ATP (pg/ml)** | **copies of 16S gene/µL** |
| **Tap 1** | **Pre-flush** | 54020 | 43993 | 60.5 | 2.03 | 201150 | 49446 | 42673 | 55.5 | 1.91 | 542150 |
|  | **Post-flush** | 210 ± 174 | 25 ± 21 | 0.72 ± 0.23 | 0.09 ± 0.05 | 1000 | 1610 ± 966 | 870 ± 401 | 0.86 ± 0.27 | n.d. | 1898 ± 1269 |
|  | **Incubated** | 30983 ± 3154 | 27800 ±1810 | 29.50 ± 26.02 | 3.63 ± 0.29 | 128482 ± 105667 | 31303 ± 15250 | 28100 ±13209 | 31.58 ± 30.30 | 8.42 ± 2.52 | 262350 ± 54589 |
| **Tap 2** | **Pre-flush** | 25000 | 16913 | 6.85 | 0.26 | 14230 | 25533 | 4860 | 4.12 | 0.41 | 40335 |
|  | **Post-flush** | 650 ± 33 | 413 ± 179 | 0.50 ± 0 | n.d. | 1132.75 ± 188 | 740 ± 94 | 383 ± 71 | 0.74 ± 0.33 | 0.23 ± 0.03 | 5267 ± 2873 |
|  | **Incubated** | 26257 ± 2560 | 22000 ± 2706 | 9.35 ± 1.63 | 5.40 ± 0.43 | 93613 ± 40641 | 22713 ± 4742 | 16337 ± 3276 | 8.10 ± 1.13 | 4.31 ± 0.13 | 260025 ± 118688 |

**Supplementary Table S6B. Significance values of the factors’ effects (tap location, group type/cell density, and its interactive effects) towards measured variables (TCC/ICC, 16S rRNA gene copies, DNA yield, ATP) and based on two-way ANOVA (with interaction effect) for controlled stagnation experiment.** *P* value < 0.05 is considered significant (denoted by numbers in bold with a *).

|  | ***P* value** | | | | |  |
| --- | --- | --- | --- | --- | --- | --- |
| **Factors** | TCC | ICC | **16S rRNA gene copies** | **DNA yield** | **ATP concentration** | |
| **Tap location** | 0.001* | 0* | 0.1 | **0.001*** | 0.27 | |
| **Sample group (cell density)** | 0* | 0* | **0.003*** | **0.0007*** | **0*** | |
| **Tap location × sample group** | 0.001* | 0* | **0.026*** | **0.004*** | 0.58 | |

**Supplementary Table S6C. Significance values of multiple comparisons between groups based on Post-hoc Tukey HSD test. *P* value < 0.05 is considered significant (denoted by numbers in bold with a *).**

|  |  | ***P* value** | | | | |
| --- | --- | --- | --- | --- | --- | --- |
| **Comparison** | | TCC | ICC | **16S rRNA gene copies** | **DNA yield** | **ATP concentration** |
| **Post flush** | **Pre-flush** | 0* | 0* | **0.014*** | **0*** | 0.48 |
| **Incubated** | **Pre-flush** | 0.005* | 0.44 | 0.97 | 0.19 | **0.0009*** |
| **Incubated** | **Post-flush** | 0* | 0* | **0.005*** | **0.008*** | **0*** |

**Supplementary Table S7A. Results of pairwise adonis/PERMANOVA for multiple comparisons of sample groups extracted with PC membrane only after data decontamination.** *P* value < 0.05 is considered significant (denoted by numbers in bold with a *).

| **pairs** | **Df** | **SumsOfSqs** | **F.Model** | **R2** | **p.value** | **p.adjusted** |
| --- | --- | --- | --- | --- | --- | --- |
| Incubated vs Pre-flush | 1 | 1.22 | 9.04 | 0.41 | 0.001 | **0.003*** |
| Incubated vs Post-flush | 1 | 1.25 | 7.93 | 0.36 | 0.001 | **0.003*** |
| Pre-flush vs Post-flush | 1 | 0.94 | 6.51 | 0.48 | 0.028 | 0.08 |

**Supplementary Table S7B. Results for pairwise adonis/PERMANOVA for multiple comparisons of different taps from controlled stagnation experiment after data decontamination.** *P* value < 0.05 is considered significant (denoted by numbers in bold with a *).

| **pairs** | **Df** | **SumsOfSqs** | **F.Model** | **R2** | **p.value** | **p.adjusted** |
| --- | --- | --- | --- | --- | --- | --- |
| Tap 1 vs Tap 2 | 1 | 0.68 | 2.96 | 0.14 | 0.013 | **0.013*** |

**Supplementary Method S1. Quantification of total bacterial 16S rRNA gene**

Quantitative PCR was performed for the measurement of total bacteria based on 16S rRNA gene copy numbers in all samples by DNASense (Denmark) according to their in-house protocol. The abundance of 16S rRNA genes per ng of isolated DNA was estimated based on the broad range qPCR probe and primer set [1]. A linearized plasmid containing the qPCR amplicon was used to create the standard curve as previously described [2]. Briefly, the forward (5’TCCTACGGGAGGCAGCAGT3’) and reverse (5’GGACTACCAGGGTATCTAATCCTGTT3’) primers were used to amplify the qPCR amplicon of from *E. coli* MG1655 using the AccuPrime Pfx DNA polymerase (Thermo Scientific). The PCR was carried out according to the recommendations of the manufacturer with the following PCR program: PCR activation (94 °C, 2 min) followed by 30 cycles of denaturation (94 °C, 30 s), annealing (60 °C, 30 s)and extension (68 °C, 90 s) and a final extension (68 °C, 5 min). The PCR product was purified on an Egel Clone ell gel (Thermo Scientific) and cloned into the pCR4TOPO vector using the TOPO TA CloningKit for Sequencing (Thermo Scientific) according to the recommendation of the manufacturer. The obtained plasmid subsequently linearized with FastDigest NcoI(Fermentas), blunted using the Klenow fragment (Fermentas). The concentration of the amplicon stock was determined using the Qubit HS dsDNA assay kit (Life Technologies) and the copy number was then calculated based on the molecular weight of the linearized plasmid. The amplicon stock was diluted to 108 copies/μL in 10 mM tris buffer (pH 8.5) and stored as aliquots at 18 °C.

Sample qPCR measurements were done in technical duplicates using the Mx3005P qPCR system (Stratagene) and the EXPRESS qPCR Supermix (Life Technologies). Reactions of 24 μL were prepared according to instructions of the manufacturer using 50 nM ROX, 500 nM of each primer, 200 nM hydrolysis probe (6FAM) 5’CGTATTACCGCGGCTGCTGGCAC3’(BHQ1), and 1 μL template DNA. The qPCR reaction conditions were as follows: UDG incubation (50 °C, 2 min) and PCR activation (95 °C, 2 min) followed by 45 cycles of denaturation (95 °C, 15 s) and combined annealing and extension (60 °C, 1 min). Amplicon standards with concentrations ranging from 10^3^-10^7^ copies/μL were included for all qPCR runs and used for quantification. A clear logarithmic correlation was found between amplicon concentration and the Cq value (R2 > 0.99) and the efficiency of the qPCR was acceptable (> 90 %). All primers and probes were HPLC purified (DNA Technology, Denmark).

**References**

1 Nadkarni MA, Martin FE, Jacques NA & Hunter N (2002) Determination of bacterial load by realtime PCR using a broadrange (universal) probe and primers set. *Microbiology (Reading, England)* 148, 257–266.

2 Karst SM, Albertsen M, Kirkegaard RH, Dueholm MS & Nielsen PH (2016) *Molecular methods* (M. C. M. van Loosdrecht, P. H. Nielsen, C. M. Lopez Vazquez,and D. Brdjanovic, eds.) IWA Publishing.
